# The orbitofrontal cortex projects to the parvafox nucleus of the ventrolateral hypothalamus and to its targets in the ventromedial periaqueductal grey matter

**DOI:** 10.1007/s00429-018-1771-5

**Published:** 2018-10-12

**Authors:** Alexandre Babalian, Simone Eichenberger, Alessandro Bilella, Franck Girard, Viktoria Szabolcsi, Diana Roccaro, Gonzalo Alvarez-Bolado, Chun Xu, Marco R. Celio

**Affiliations:** 1 Anatomy and Programme in Neuroscience, Faculty of Science and Medicine, University of Fribourg, Rte. A. Gockel 1, 1700 Fribourg, Switzerland; 2 Institute of Anatomy and Cell Biology, University of Heidelberg, im Neuenheimer Feld 307, 69120 Heidelberg, Germany; 3 Friedrich Miescher Institute, Maulbeerstrasse 66, 4058 Basel, Switzerland

**Keywords:** PV1, Viral tracers, Su3, PV2, Somatic marker

## Abstract

Although connections between the orbitofrontal cortex (OFC)—the seat of high cognitive functions—the lateral hypothalamus and the periaqueductal grey (PAG) have been recognized in the past, the precise targets of the descending fibres have not been identified. In the present study, viral tracer-transport experiments revealed neurons of the lateral (LO) and the ventrolateral (VLO) OFC (homologous to part of Area 13 in primates) to project to a circumscribed region in the ventrolateral hypothalamus, namely, the horizontally oriented, cylindrical parvalbumin- and Foxb1-expressing (parvafox) nucleus. The fine collaterals stem from coarse axons in the internal capsule and form excitatory synapses specifically with neurons of the parvafox nucleus, avoiding the rest of the hypothalamus. In its further caudal course, this contingent of LO/VLO-axons projects collaterals to the Su3- and the PV2 nuclei, which lie ventral to the aqueduct in the (PAG), where the terminals fields overlap those deriving from the parvafox nucleus itself. The targeting of the parvafox nucleus by the LO/VLO-projections, and the overlapping of their terminal fields within the PAG, suggest that the two cerebral sites interact closely. An involvement of this LO/VLO-driven circuit in the somatic manifestation of behavioural events is conceivable.

**Electronic supplementary material:**

The online version of this article (10.1007/s00429-018-1771-5) contains supplementary material, which is available to authorized users.

## Introduction

In a series of magisterial papers, Joseph Price and his colleagues characterized the frontal cortex and its projections in various species (Bacon and Smith 1993; Carmichael and Price 1994, 1996; Ongur et al. 2003). In the rat, the OFC was subdivided—medially-to-laterally—into ventral (VO), ventrolateral (VLO) and lateral (LO) regions (Krettek and Price 1977), which are analogous to the multimodal areas 14, 13a and 13 m/l, respectively, in the monkey (Price 2007). Area 13a, the so-called “visceromotor” region, is a constituent of the medial network (Carmichael and Price 1996), receiving inputs from limbic structures and projecting to the hypothalamus and the PAG. Area 13 m/l forms a part of the orbital network (Carmichael and Price 1996). Receiving multimodal information, it is consequently referred to as the “viscerosensory” region. It projects almost no descending axons to either the amygdala, the hypothalamus or the PAG (Ongur et al. 1998).

The LO/VLO cortex in rodents is therefore a homologue of part of the reward network region in the ventromedial prefrontal cortex (VM) (Ongur and Price 2000) that is involved in decision-making and emotional handling in primates (Bechara et al. 2000). In humans, lesioning of the VM interferes with the processing of somatic or emotional signals, thereby leading to an impairment in decision-making (Bechara et al. 2000). An influential hypothesis (“somatic marker”) postulates the VM to be a key link between emotional regions (amygdala) and the autonomic nervous system (Damasio 1996), of which the hypothalamus is the key organizer.

The projections from the OFC in rodents have been exhaustively investigated by various authors by means of antero- and retrograde tracing, The findings have revealed the OFC to be connected with the primary olfactory cortex (Price 1985), the piriform cortex (Illig 2005), the caudatoputamen (Beckstead 1979; Berendse et al. 1992; Gabbott et al. 2005; Groenewegen et al. 1990; Leonard 1969; Schilman et al. 2008), the amygdala (Groenewegen et al. 1990; McDonald et al. 1996), the extended amygdala (Groenewegen et al. 1990; Reynolds and Zahm 2005), the submedial (Coffield et al. 1992; Craig et al. 1982; Price and Slotnick 1983; Yoshida et al. 1992) and the mediodorsal thalamic nuclei (Beckstead 1979; Gabbott et al. 2005; Groenewegen 1988; Guldin et al. 1981; Leonard 1969; Price and Slotnick 1983; Ray and Price 1993; Reep et al. 1996), the parafascicular nucleus (Jones and Leavitt 1974), the claustrum (Zhang et al. 2001), the lateral hypothalamus (Gabbott et al. 2005; Hardy 1994; Hurley et al. 1991; Price et al. 1991), the PAG (Hardy 1986; Wyss and Sripanidkulchai 1984), the ventrolateral PAG (Beckstead 1979; Craig et al. 1982; Leonard 1969) and the oculomotor complex (Leichnetz and Gonzalo-Ruiz 1987b; Leichnetz et al. 1987a). The VLO-cortex projects additionally to the visual cortex (Reep et al. 1996). The targets of OFC projections are distributed throughout the entire brain—the hippocampus and the cerebellum exempted. Although the lateral hypothalamus and the periaqueductal grey matter (PAG) have been shown to receive terminals, their localization has not been identified with precision.

Using viral constructs (Chamberlin et al. 1998; Wickersham et al. 2007a) and genetically modified mice that express Cre-recombinase in parvalbumin- (Parv) (Hippenmeyer et al. 2005) and/or Foxb1-expressing neurons (Zhao et al. 2007), we demonstrate that the LO- and the VLO-cortices heavily project collaterals to three as yet less well-known structures, namely, the parvafox nucleus (Bilella et al. 2014; Celio 1990; Celio et al. 2013; Girard et al. 2011; Meszar et al. 2012), the supraoculomotor nucleus (Su3) (Carrive and Paxinos 1994) and the parvalbumin 2 nucleus (PV2) (Celio et al. 2013) of the PAG. The parvafox nucleus [formerly called PV1 nucleus (Celio 1990)], is located amongst the fibres of the medial forebrain bundle in the ventrolateral hypothalamus. The Su3 and the PV2 nuclei are two longitudinally oriented columns of neurons, which are located ventral to the aqueduct at the border of the PAG in the mesencephalic tegmentum.

Although the largest contingent of axon terminals derives from pyramidal cells in layer V–VI of the LO/VLO-cortex and is excitatory, parvalbumin-expressing and a few GABAergic neurons also contribute to the projections. By virtue of its dual inhibitory and excitatory projections, the LO/VLO-cortex may modulate the activity of the parvafox nucleus and its PAG targets.

## Materials and methods

This study was conducted in accordance with the regulations of the Swiss Federal Animal Protection Law and under the supervision of the Veterinary Authority of the Canton of Fribourg (permissions 2013-04-Fr; 2013-05-FR, 2016-36-Fr).

Experiments were performed on 36 adult C57/Bl6 mice and 13 Wistar albino rats (Janvier, Lyon, France) of both genders, weighing 26–39 g and 240–325 g, respectively (Table 1). Other strains of mice that were used were either homozygous for the *Pvalb*-Cre genotype [129P2-*Pvalb* < tm1(cre)Arbr>/J] (Hippenmeyer et al. 2005) or heterozygous for the *Foxb1*-Cre one [Foxb1^*tm1cre*−*EGFPGabo*^] (Alvarez-Bolado et al. 2000) (Table 1). *TVA*-floxed mice [B6;129P2-*Gt(ROSA)26Sor*^*tm1(CAG*−*RABVgp4*,−*TVA)Arenk*^/J, strain 024708] were used in the rabies experiments and *VGAT*-Cre mice [*Slc32a1*^*tm2(cre)Low l*^, strain 016962 (Jackson Laboratory, Bar Harbor, Maine, USA)] for anterograde tracing with Cre-dependent constructs. The mice that were used for the trans-synaptic rabies injections were of the TVA-*Pvalb*/*Foxb*1 genotype; they were bred in house (Table 1).

**Table 1:** Strains of rats and mice that were utilized to study the projections from the OFC

Name	Line	Strain Nr. (Jackson)	Source
Wild type	Wistar		Janvier, Lyon, France
Wild type	C57/Bl6		Janvier, Lyon, France
*Pvalb*-Cre	129P2-*Pvalb*^tm1(cre)Arbr^/J	008069	Dr. Silvia Arber, Basel (Switzerland) and Jackson Laboratory
*Foxb1-*Cre	Foxb1^*tm1cre*−*EGFPGabo*^		Dr. Gonzalo Alvarez-Bolado Heidelberg, Germany
TVA-floxed mice	B6;129P2-*Gt(ROSA)26Sor*^*tm1(CAG*−*RABVgp4*,−*TVA)Arenk*^/J	024708	Jackson Laboratory
VGAT-Cre	Slc32a1 ^tm2(cre)Low l^	016962	Jackson Laboratory
*TVA*-*Pvalb*-Cre/*Foxb*1-Cre	*TVA*-*Pvalb*-Cre/*Foxb1*-Cre		Bred in house

To check for the presence of projecting parvalbumin-expressing neurons, Cre-dependent tracers were also injected into the OFC of *Pvalb*-Cre mice. The *Pvalb*-Cre and the *Foxb1-*Cre mice were used in the co-labelling experiments: the non-Cre-dependent tracers were injected into the OFC and the Cre-dependent ones, labelled with another fluorescence-dye, into the parvafox nucleus. The TVA-floxed mice, and the *Pvalb*-Cre/*Foxb1-*Cre ones with which they were bred, were employed for the injection of Cre-dependent rabies-tracers into the parvafox nucleus, with a view to studying the trans-synaptic location of the retrogradely labelled neurons in the OFC

The animals were anaesthetized with a mixture of ketamine (40–60 mg/kg of body weight) and xylazine (10–15 mg/kg of body weight) which was diluted with physiological (0.9%) saline. If necessary, supplementary, lower (1/4–1/3) doses of the anaesthetic were administered during the stereotactic procedure, if any signs of awakening became manifest.

### Anterograde tracing experiments (Table 2a)

**Table 2:** List of the antero- and retrograde tracers that were used for the experiments.

a: Anterograde tracers			
		Species	Source
Viral, non-Cre-dependent			
1	AAV2/1.hSynapsin.EGFP.WPRE.bGH	Rats and mice	Vector Core, University of North Carolina, USA
2	AAV1.hSynapsin.TurboRFP.WPRE.rBG	Rat	Vector Core, University of North Carolina, USA
3	AAV9.hSynapsin.TurboRFP.WPRE.rBG	Rat	Vector Core, University of Pennsylvania, USA
Viral, Cre-dependent			
4	AAV2/1.CAG.FLEX.EGFP.WPRE.bGH	Mouse	Vector Core, University of Pennsylvania, USA
5	AAV1.CAG.flex.tdTomato.WPRE.bGH		Vector Core, University of Pennsylvania, USA
Non-viral			
6	Biotinylated dextran (MW 10′000) anterograde tracer	Mouse	*Invitrogen* (D1956 Lot 1148353)

b: Retrograde tracers		
		Source
Trans-synaptic transport (rabies)		
7	rAAV8/CA-Flex-RG.ape	Vector Core, University of North Carolina (USA)
8	rAAV5/EF1-Flex-TVA-Cherry.ape	Vector Core, University of North Carolina (USA)
	Env-A Δ-G rabies-EGFP	Friedrich Miescher Institute-Basel (Switzerland)
Others		
10	Biotinylated dextran (BDA, MW 3000) retrograde tracer	Invitrogen (D7135), Waltham, USA
11	Fluorogold Anti-fluorogold antiserum	Fluorogold, Denver, USA Millipore AB153 Lot 2161122, USA

The anterograde, non-Cre-dependent tracers were injected primarily into the OFC, and the Cre-dependent ones into the parvafox nucleus of *Pvalb*-Cre, *Foxb1-*Cre or *Pvalb*-Cre/*Foxb1-*Cre mice. The classical retrograde tracers (BDA, Fluorogold) were injected into the targets of the OFC projection, namely, into the parvafox nucleus and the PAG, to reveal the presence of double-stained neurons in the OFC

The head of the animal was secured in the stereotaxic apparatus (Kopf Model 5000) and a craniotomy was performed over the target region in the orbital cortex. Tracers 1–6 in Table 2a were used as anterograde tracers in these experiments.

#### Injections in the OFC

The tracers were injected via a fine-bored needle (external diameter: 0.14 mm, GA: 34), which was connected to a 2.5-µl Hamilton syringe that was mounted on a manual microinjection unit (Kopf, model 5000). In rats, the injections were made at different sites of the LO around central coordinates of anteroposterior (AP): + 4.2, mediolateral (ML): ± 2.4, dorsoventral (DV): − 3.5 (in mm, with respect to the bregma level and the brain surface). If not otherwise specified elsewhere, the injections in mice were usually made around the stereotaxic coordinates of AP: + 2.8, ML: ± 1.3, DV: − 1.8. 20–80 nl of the tracer (Table 2a) was injected into rats and 15–20 nl into mice during an interval of 0.5–1 min. After the injection, the needle was left in place for 3–5 min to allow the tracer to diffuse at the injection site. The needle was then withdrawn, the skin over the skull was sutured and the animals were left to recover. In most of the experiments with rats, bilateral injections of the same or different tracers were made at various AP and ML coordinates. The position of the needle was varied such that deposits of label involved in entirety the medial, lateral and insular portions of the dorsal sulcal cortex (MO, VO, VLO, LO, DLO, AIV). Due to the intricacy and the small size of the various orbitofrontal regions, injections were almost never confined to one region alone and tracers often suffused adjacent cortical areas.

#### A note on the OFC tracing data found in the Allen Brain Atlas (ABA)

After its first appearance in 2013, subsequent editions of the Allen Brain Atlas (ABA) have reported an ever-increasing body of data that have been garnered from the stereotactic injection of viral tracers into the OFC of murine brains (suppl. Table. 1). We drew on some of the self-same viral tracers in our own experiments. The data that are presented in the ABA are based upon the implementation of an iontophoretic technique, which involves the injection of a viral tracer at two levels in the cortex, whence it attains all layers. The volume of the injected tissue is then calculated. In our experiments, a defined volume of the viral tracer was delivered in a single injection via a Hamilton syringe to the targeted site. Since our data accord well with those that are presented in the ABA, we presume that the different modes of delivery of the viral tracers (iontophoresis versus microinjection) had no impact on their accessibility to nerve cells.

#### Injections in the parvafox nucleus

For the injections into the parvafox nucleus of mice, the needle was positioned at bregma level: − 1.5 mm, ML: 1.3 mm, DV: 4.9–5.1 mm (Bilella et al. 2016).

#### Double injection of anterograde tracers in the OFC and in the parvafox nucleus

A red anterograde tracer was injected in the OFC and a green one in the parvafox nucleus (or vice-versa). In these specimens, we investigated the spatial relationship between the OFC endings and the endings from the parvafox nucleus in the Su3 and the PV2 regions of the PAG. A Cre-dependent red-construct was injected into the parvafox nucleus and a Cre-dependent (or non-Cre-dependent) GFP-tracer into the LO/-VLO-cortex. Eight PV-Cre (552-12, 553-12, 138-13, 222-13, 223-13, 357-14, 394-14, 395-14), three Foxb1-Cre (209-14, 223-14, 390-14) mice, and one PV-Cre/Foxb1-Cre (223-14) mouse were investigated.

#### Are subpopulations of the OFC-cells projecting to the parvafox nucleus inhibitory or Parv-expressing (Lee et al. 2014)?

A Cre-dependent tracer was injected in the OFC of *VGAT*-Cre or *PValb*-Cre mice to detect terminals on neurons of the parvafox nucleus and in the PAG. The animals used for these experiments were six *Pvalb*-Cre mice (356-14; 357-14; 394-14; 306-15; 307-15; 308-15) and three *VGAT*-Cre mice (234-16, 235-16, 236-16).

#### Do experiments with classical tracers confirm the results found with viral tracers?

In eight mice (250-13, 399-15, 400-15, 401-15, 402-15; 28-16, 29-16), biotinylated dextran (BDA 10,000 MW, Invitrogen, USA) was injected at various medio-lateral coordinates into the OFC. The distribution of the terminals in the parvafox nucleus and in the PAG corresponded exactly to the picture that was revealed after the injection of the viral tracers. Only injections that involved the LO/VLO-cortex disclosed the typical “pony-tail-like” terminal field in the parvafox nucleus.

The cortices at the medial and lateral boundaries of the orbital cortex were selectively targeted in various experiments (126-13 [prelimbic (PrL), medial orbital (MO)]; 185-13 [MO]; 186-13 [MO]; 130-15 [PrL]; 132-15 [infralimbic (IL)]; 552-12 [DLO]; 553-12 [DLO]; 556-12 [DLO]). None revealed targeted terminals in the parvafox- or in the Su3 and PV2-nuclei, only a diffuse innervation of the lateral hypothalamus and of other columns in the PAG. As demonstrated in the ABA-database, tracer injection in the AID, AIV and AIP did not show selective innervation neither of the parvafox nor of Su3 and PV2-nuclei (http://connectivity.brain-map.org/).

In a few experiments, the injection needles pierced the OFC and penetrated the olfactory bulb (394-13, 395-13), the anterior olfactory nucleus (132-15; 309-15), the olfactory tract (386-13) and the olfactory tuberculum (386-13).

### Retrograde tracing experiments (Table 2b)

To confirm the existence of the projections that were observed in the anterograde tracing experiments and to ascertain whether the projections to the different sites originate from the same or different populations of neurons, retrograde (including double) labelling experiments were performed (tracers 7–11 in Table 2b). These included also some double-labelling experiments targeting in the same animal the PAG and the parvafox (both targets of the OFC as the present study reveals). Two classical retrograde tracers, viz. Fluorogold [2% in physiological (0.9%) saline; 12–30 nl/1930s] and biotinylated dextran amine [(BDA) 10% in physiological (0.9%) saline; 40–100 nl/1–2 min] were injected into targeted regions of the LO/VLO-projections in mice, namely, into the PAG (coordinates of AP: − 4.1, ML:± 0.5, DV: − 2.7) and/or the parvafox nucleus (coordinates of AP: − 1.5, ML: ± 1.3, DV: − 4.9) using the same tools and procedures that are described above for the injection of the anterograde tracers.

In addition, trans-synaptic labelling of the parvafox nucleus was executed in *Pvalb*-Cre/*Foxb1*-Cre mice that had been bred with TVA-floxed (*Pvalb*-*Foxb1*-*TVA*) mice. To this end, a Cre-dependent glycoprotein-deleted mutant strain of the SAD B19 rabies vaccine strain bearing an EGFP-insert (Env-A Δ-G rabies-EGFP) was concomitantly injected with AAV-Flex-G into the parvafox nucleus (Table 2b) (Wickersham et al. 2007a).

### Histological procedures

After 6–8 days for retrograde, and 3–4 weeks for anterograde tracing experiments, deep anaesthesia was induced in the animals by the administration of a lethal dose of pentobarbital (150–200 mg/kg of body weight). They were then transcardially perfused, first with physiological (0.9%) saline and then with paraformaldehyde (4% in 0.1 M phosphate buffer, pH 7.4) the brains were excised and postfixed overnight in 4% paraformaldehyde. They were then submerged in a 30% solution of sucrose for cryo-protection. Using a cryomobile (Reichert-Jung), the brains were serially sliced into 40/80-µm-thick sections, which were collected in 0.1 M phosphate buffer containing 0.01% sodium azide. The sections were usually cut in the coronal (frontal) plane. However, in four brains (393-13, 142-14, 132-15, 307-15) the sections were prepared in the sagittal plane, and in three (386-13, 394-13, 141-14) they were cut in the horizontal direction. The sections were subsequently incubated with the appropriate antibodies to confirm the precision of the injection and the presence of neuronal endings in the region of interest, particularly around the cells of the parvafox nucleus. Series of sections that were derived from untreated mice and rats were stained with Nissl and exposed to an antibody against non-phosphorylated neurofilaments (SMI-32), which served as a neuronal marker (Franklin and Chudasama 2012) to define the subdivision of the prefrontal cortex. The sections were mounted on glass slides for histological inspection in either a Leica 6000 epifluorescence microscope [equipped with a Hamamatsu C4742-95 camera], a digital slide-scanner (Nanozoomer, Hamamatsu), or a Leica TCS SP5 confocal laser microscope. Staining with GFP, RFP, Tomato or Fluorogold was detected by virtue of the intrinsic fluorescence; that with BDA was revealed after exposure to streptavidin-Cy3.

### Immunohistochemistry

The immunofluorescence technique and the immunoperoxidase reaction were performed as previously described (Gerig and Celio 2007; Meszar et al. 2012). In short, floating sections were incubated in 24-well plates with the primary antisera or antibodies, which were diluted in the range 1:1000–1: 5000 (Table 3). The efficacy of the antisera and the antibodies against Parv had been hitherto established in antigen-pre-adsorption experiments, by immunoblotting and by the absence of immunoreactivity in knock-out mice (see Table 3). Incubation with the biotinylated secondary antibody was followed by exposure to either streptavidin-Cy2 (Alexa460), -Cy3 (Alexa 550) or -Cy5 (Alexa 650).

**Table 3:** List of the antibodies and the antisera that were used in the experiments

Antibody	Antigen	Source	Species	Dilution
PV235	Purified carp parvalbumin	Swant Inc., Marly, Switzerland	Mouse monoclonal Lot 10-11F	1:1000–5000
PV25	Recombinant rat parvalbumin	Swant Inc., Marly, Switzerland	Rabbit polyclonal Lot 5.10	1:1000–1:5000
GP72	Recombinant mouse parvalbumin	Swant Inc., Marly, Switzerland	Guinea pig polyclonal	1:1000–1:5000
PVG213/214	Recombinant rat parvalbumin	Swant Inc., Marly, Switzerland	Goat polyclonal	1:1000
GFP	Recombinant peptide	Molecular Probes, Waltham, (USA)	Rabbit polyclonal	1:3000
5-HT	Serotonin	Immunonuclear	Rabbit polyclonal	1:2000
TH	Tyrosine-hydroxylase	Immunostar, Stillwater (USA)	Rabbit polyclonal	1:10,000
SMI-32	Non-phosphorylated filaments	Millipore, USA	Mouse monoclonal	1:1000
VGlut 1	Recombinant peptide	Synaptic System, Germany	Rabbit or mouse	1:5000/1:20,000
VGlut 2	Recombinant peptide	Synaptic System, Germany	Rabbit	1:10,000
GAD	Recombinant peptide	Millipore, USA	Mouse	1:2000

The antibodies against Parv were utilized primarily to confirm that the endings from the OFC did indeed impinge on parvalbumin-immunoreactive neurons in the parvafox nucleus. The serotonin- and TH-antisera helped to define the borders of the raphe and the coeruleus nuclei. GFP-antisera served to enhance the fluorescence in the terminals of the brainstem and the spinal cord

### Electron microscopy

In one case (127-13) five coronal sections of the region of the hypothalamus in which positive terminals were observed around the parvafox nucleus were incubated with antibodies against GFP during 5 days (without Triton-X100). The immunostaining was continued with a biotinylated antiserum against rabbit-IgG (1 day) and followed by avidin-peroxidase (1 day). After washing, the enzymatic activity was revealed by incubating the sections with DAB-H2O2. The sections were further post-fixed with 2.5% glutaraldehyde in 0.1 M cacodylate buffer, pH 7.3 and after washing, were exposed to 1% OsO4 (osmium tetroxide) in phosphate buffer for 2 h. Embedding took place in Epon. Semi- (0.5 µm) and ultrathin sections (60 nm, grey interference colour) were cut with a Reichert Ultramicrotome and mounted on one-hole grids. Uranyl acetate and lead citrate were employed for contrasting purposes. The region of the parvafox was searched for the presence of synapses between immunoreactive terminals and dendrites or cell bodies using a Jeol microscope.

## Results

### Delimitation of the OFC and the injection sites

**Fig. 1:**
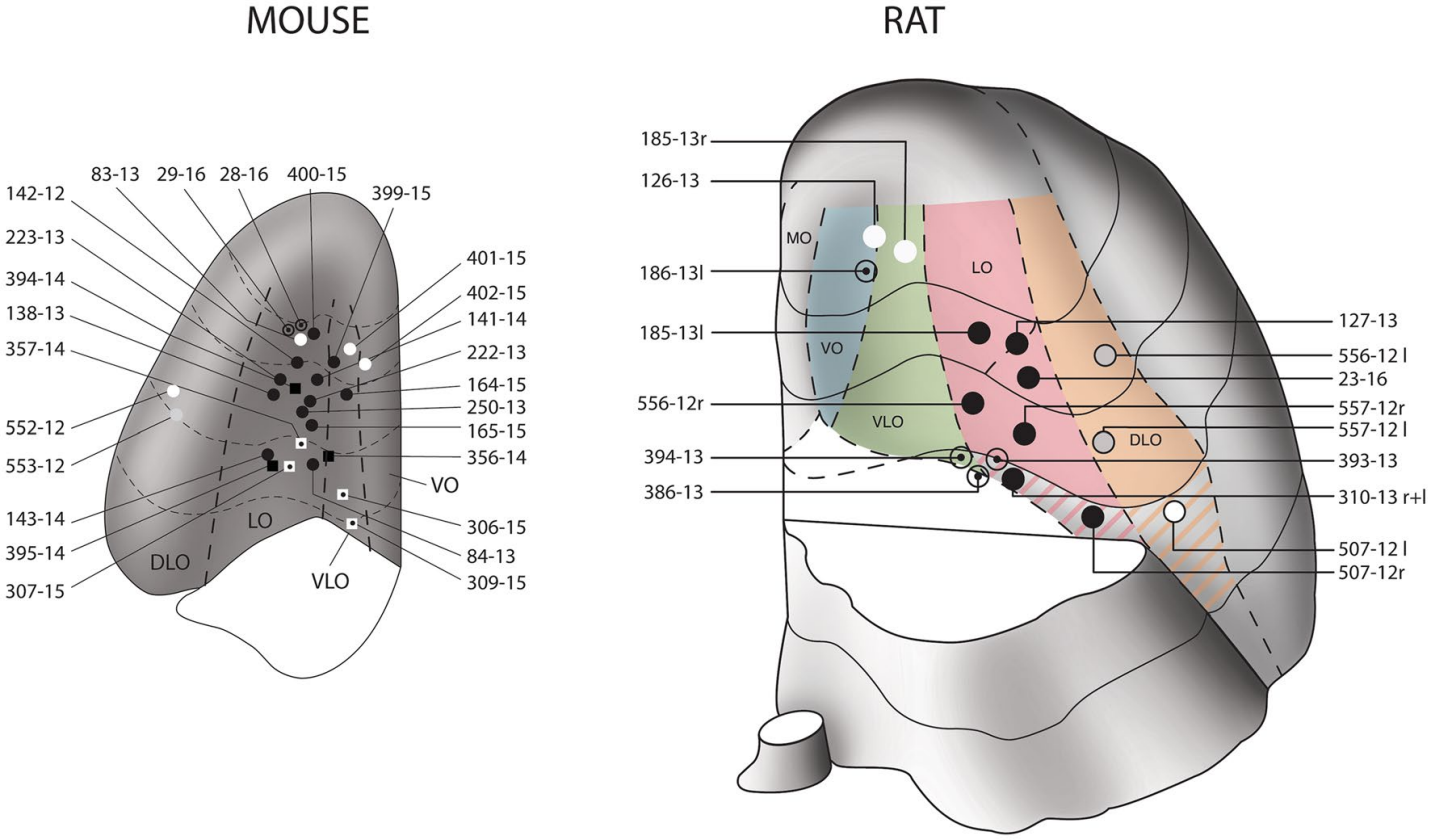
Map of the injections sites. Schematic drawings of the positions of the needle tip in the stereotactic injections into the OFC of 26 mice (left) and 12 rats (right, 4 bilateral). The drawings depict the basal surface of the OFC after removing the olfactory bulb and the piriform region. The OFC is subdivided into four longitudinal regions: medial (MO), ventrolateral (VLO), lateral (LO) and dorsolateral (DLO), according to (Dong 2008; Price 2007). The injections into mice were located in the central LO/VLO portion, those into rats in various other parts of the OFC. The black circles indicate the positions of injections that revealed terminals in the parvafox nucleus of the ventrolateral hypothalamus as well as those in the Su3- and the PV2 nuclei of the PAG. The circles with a central point indicate the positions of injections that revealed terminals in the parvafox nucleus of the ventrolateral hypothalamus alone, not those in the Su3- and the PV2 nuclei. The grey circles indicate the positions of injections that revealed terminals in the Su3- and the PV2 nuclei of the PAG, not those in the parvafox nucleus of the ventrolateral hypothalamus. The white circles indicate the positions of injections that did not reveal terminals either in the parvafox nucleus or in the Su3- and the PV2 nuclei of the PAG. The squares indicate the positions of injections into the OFC of *Pvalb*-Cre- or *Foxb1*-Cre-mice. The colour-coding of the squares corresponds to that of the circles. The OFC-subdivision of the mouse brain was based upon information that was derived from cresyl violet-stained and SMI-31 immunostained stained serial sections through the brains of wild-type mice (C57/B16). The right-hand picture is a modified version of a published figure (Groenewegen 1988)

**Fig. 2:**
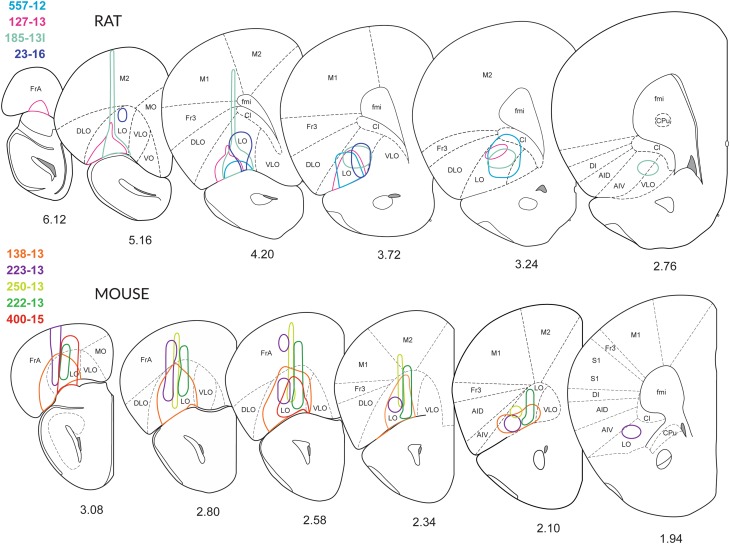
Extent of the injections. Schematic drawings of coronal sections of the right hemisphere depicting the extent of the injection sites through the brains of four rats (557-12, 127-13, 158-13 and 23-16) and five mice (138-13, 222-13, 223-13, 250-13 and 400-15), reproduced from (Franklin and Paxinos 2008). The medial boundary of the LO-cortex has been slightly modified, and the VLO-cortex has been introduced according to (Dong 2008; Krettek and Price 1977). In most cases, an adeno-associated virus tracer was injected stereotactically. In cases 250-13 and 400-15, BDA was applied. Although the tracers were injected into the LO-cortex, also dorsal (Fr, Cl and M2) or adjacent regions (DLO and AIV) were sometimes co-labelled. In rats, the injections 557-12 and 127-13 were located mainly in the LO-cortex. In the murine OFC, the injections in the LO-cortex transgressed the border to surrounding areas. In both rats and mice, the tracer injections revealed clearly visible terminal fields in the parvafox-, the Su3- and the PV2 nuclei. The drawings were prepared based on the intrinsic fluorescence of the tracers, in the absence of amplification with antibodies. The drawings are not to scale

**Fig. 3:**
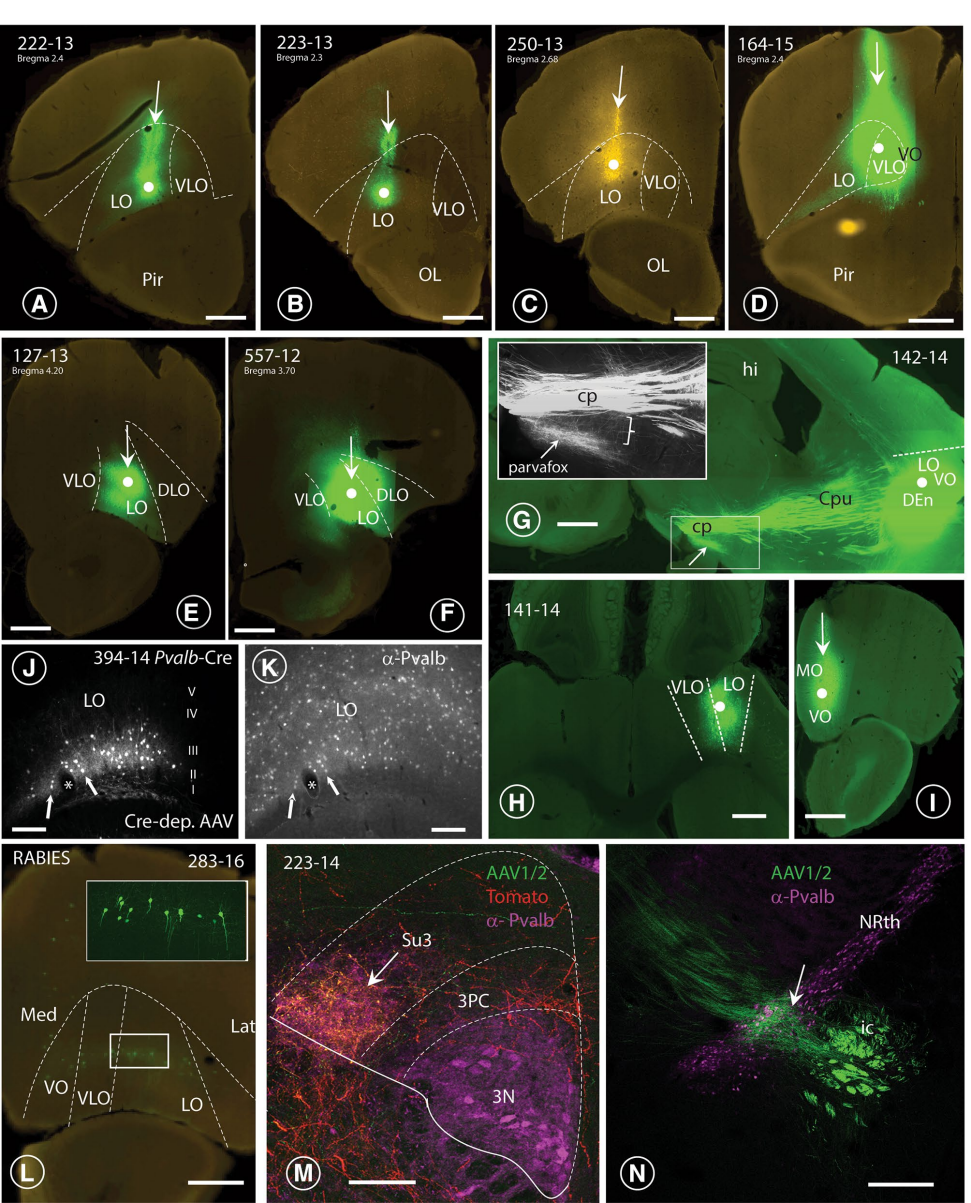
Injection sites in the OFC and terminal fields in lateral hypothalamus and PAG. **A**–**F** Nanozoomer scans of coronal sections through six specimens (4 mice: **A**–**D**; 2 rats: **E, F**), revealing the course of the Hamilton-syringe needle (vertical arrows) and the site of deposition of the tracer at its tip (white dot). In three cases (**A** 222-13; **E** 127-13; **F** 164-15; **G** 557-12), the six layers of the OFC-cortex were imbibed with the tracer, whereas in cases **B** (223-13) and **c** (250-13), only the deeper layers (V and VI) of the LO-cortex were labelled. In each of the depicted cases, axonal terminals were revealed in the parvafox nucleus, as well as in the Su3- and the PV2 nuclei. G: Para-sagittal section through the brain of a mouse in which the tracer had been injected into the LO-cortex [with spreading to the VLO- and the VO- cortices (142-14)]. The bundles of axons passing through the CPu and converging on the cerebral peduncle (cp) of the posterior hypothalamus are clearly visible (see also Fig. 4a). The terminal field to the parvafox nucleus is indicated with an arrow. The inset shows the almost vertical projection of thin collateral axons (bracket), deriving from the cp and generating the rich terminal field of the parvafox nucleus. **H** Horizontal section through a mouse brain (rostral side up), injected into the LO-with spreading to the VLO-cortex (141-14). **I** Coronal section through a mouse brain showing an injection limited to the MO-cortex (129-15), with no projections to parvafox, Su3 and PV2. **J, K** Sections through the LO-cortex in a *Pvalb*-Cre mouse (394-14) which had been injected with a Cre-dependent AAV-virus (**J**). The section was then incubated with an antibody against Parv (**K**). The cells that took up the tracer are Parv-immunoreactive (arrows). *Blood vessel. **L** Transsynaptic retrograde visualization of neurons in the prefrontal cortex after Cre-dependent rabies injection in the parvafox nucleus of a *Pvalb*-Cre/*Foxb1-*Cre mouse (183-16). Positive neurons are mainly detected in layers V–VI of the LO/VLO cortex. The inset shows an image stack taken with the confocal microscope in the area of the VLO-LO-cortex (frame): the perikarya and the apical dendrites of the pyramidal cells are well visible. *Lat* lateral, *Med* medial. **M** Overlapping terminal fields in the ventrolateral portion of the Su3-region of the PAG (arrow). The green (tracer injected in the LO-cortex) and the red (tracer injected in the parvafox of a *Pvalb*-Cre/*Foxb1-*Cre mouse) terminals intermingle and generate the orange tonality (arrow) in Su3. Pvalb-immunoreactivity (magenta) highlights the oculomotor nucleus (3N). 3PC: parvicellular part of the oculomotor nucleus. **N** Presence of terminals coming from the LO/VLO cortex in the reticular thalamic nucleus (NRth). Scale bars **A**–**D, E, H, I, L, N**: 0.5 mm;** J**,** K** 0.05 mm;** F**,** G** 1 mm;** M** 0.1 mm

Although the structural organization of the murine prefrontal cortex is assumed to be similar to that in the rat (Franklin and Chudasama 2012), the presence of a VLO-cortex between the LO and the VO in mice is debated (Franklin and Chudasama 2012). The extension of the LO-cortex is revealed after exposure to an antibody against SMI-32, which is a marker of a non-phosphorylated neurofilament subunit in rats (Franklin and Chudasama 2012) and mice (not shown). On the basis of immunoreactivity for SMI-32, we tentatively identified and delimited the VLO-region in our specimens (Figs. 1, 2 ,3) [see also (Dong 2008)].

**Fig. 4:**
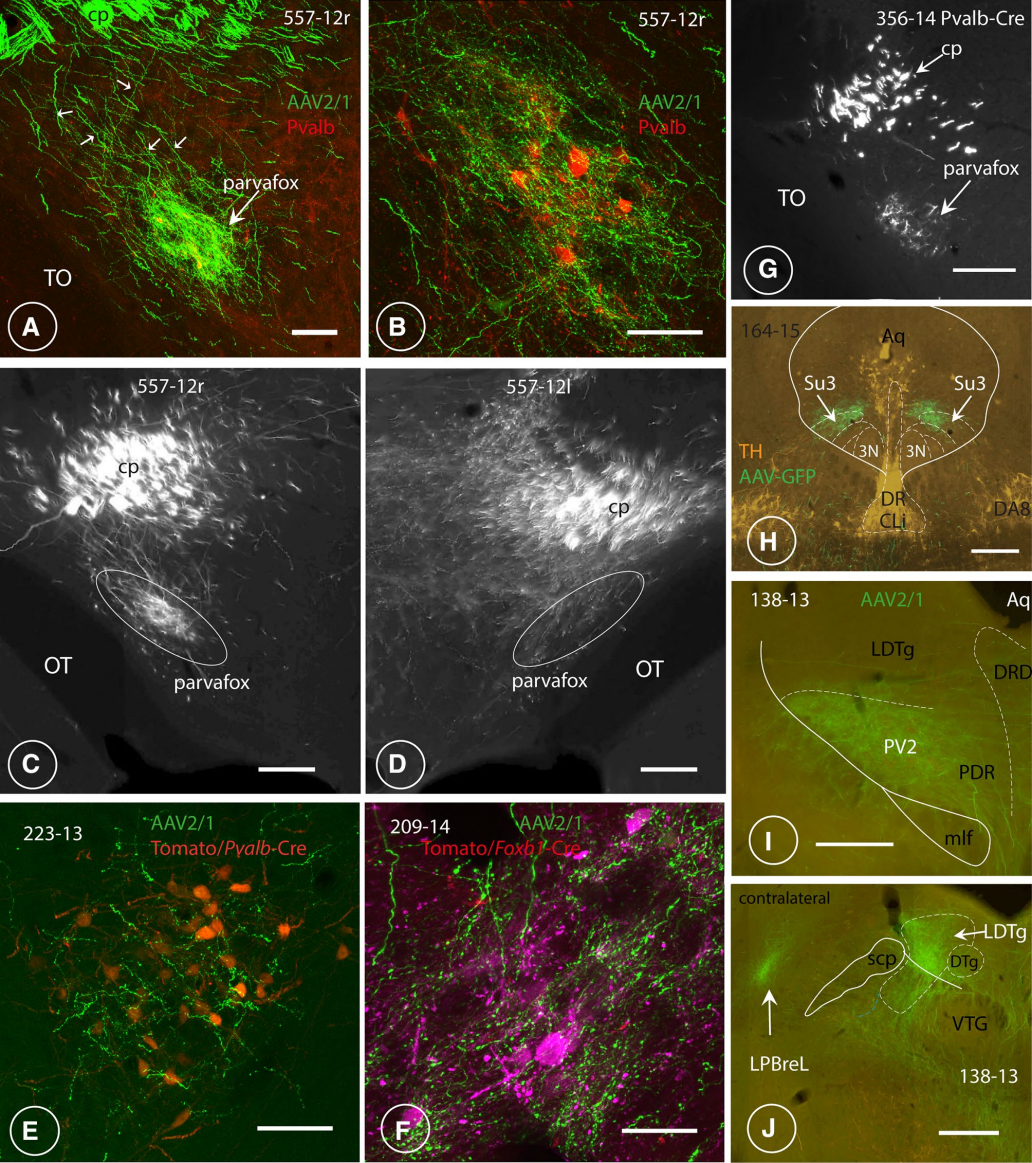
Axonal endings in the ventrolateral hypothalamus and the ventrolateral PAG. Terminals of LO/VLO-cortex-derived axons in the region of the parvafox nucleus of the ventrolateral hypothalamus and in the Su3 and PV2 nucleus of the PAG. **A, B** Low- and higher magnification views of the endings in the parvafox nucleus of rat specimen 557-12, taken at two different coronal levels. A group of thin axons (small white arrows) derives from thicker ones that are located in the cerebral peduncle (cp) and converge towards the parvalbumin-immunoreactive neurons (red, in **B**) which define the axis of the parvafox nucleus. The terminal field embraces a larger area than is occupied by the parvalbumin-expressing neurons (**B**), since the axons project also to the co-axially located *Foxb1-*expressing ones, which are located peripherally in the parvafox nucleus (Bilella et al. 2014). The parvafox nucleus is rich in terminals, some of which lie adjacent to the cell bodies and the dendrites of the parvalbumin-expressing neurons (**B**). The rest of the lateral hypothalamus receives almost no inputs. TO: optic tract Scale bars: **A** 0.1 mm; **B** 0.05 mm. **C, D** Coronal views of the parvafox nucleus and the surrounding lateral hypothalamus on both sides of the same specimen (557-12 right and left). **C** (557-12r), terminals are apparent almost exclusively in the parvafox nucleus (traced in white). **D** (557-12 l), the entire lateral hypothalamus is richly supplied with terminals. The injection (**C**) was located in the LO, the injections (**D**) were located more laterally (DLO). TO: optic tract. Scale bars: 0.2 mm. **E** LO-cortex-derived axonal endings around Parv-neurons of the parvafox nucleus in a *Pvalb*-Cre mouse that was injected stereotactically with the AAV-tomato tracer (223-13). Scale bar: 0.5 mm. **F** LO-cortex derived axonal endings around Foxb1 neurons of a Foxb1 mouse that was injected stereotactically with the tomato tracer (specimen 390-14). Scale bar: 0.5 mm. **G** LO-cortex-derived terminals in the parvafox nucleus (arrow) of a *Pvalb*-Cre mouse (specimen 356-14). The terminals in the parvafox nucleus stem from thin axons that emanate from thicker ones in the cerebral peduncle (cpd). TO: optic tract. Scale bar: 0.2 mm. **H** Topographic relationship of the OFC endings in the Su3 region and catecholaminergic neurons revealed by TH-immunofluorescence. *DR* dorsal raphe, *CLi* caudal linear raphe nucleus, *3N* oculomotor nucleus, *DA8* DA8 dopamine cells. Scale bar: 0.4 mm. **I** LO-cortex derived axonal endings around the Parv-positive neurons of the PV2-nucleus in the posterodorsal raphe nucleus. Scale bar: 0.3 mm. **J** VLO-cortex derived axons terminate in the lateral parabrachial nucleus (LPBreL) of the contralateral side. DTg, VTG: dorsal, resp. ventral tegmental nucleus. *LDTg* laterodorsal tegmental nucleus, *scp* superior cerebellar peduncle. Scale bar: 0.3 mm

By adopting the subdivision of the prefrontal cortex in rats that was introduced by the groups of Price (Price 2007) and Reep (Reep et al. 1996), most of the successful injections that revealed the presence of terminals in the parvafox nucleus were located in the LO- and the VLO-cortices. After the injection of viral tracers into the mid portion of the LO- and the VLO-cortex in rats (Figs. 1, 2, 3E, F), and in mice (Fig. 1, 2, 3A–D, G–J), enriched terminal staining was revealed in the parvafox nucleus (Fig. 4A–G). Injections in the most rostral and caudal position of the LO/VLO cortex often gave negative results (Fig. 1; see also specimen 180673746 in the ABA-database, Suppl Table 1). The injected tracer tended to spread in a rostrocaudal, longitudinal direction (not shown), as if the intrinsic texture of the OFC facilitated this path of diffusion. A strong and selective projection to the parvafox nucleus was disclosed only if the viral tracer impregnated the deep cortical layers (V and VI). Injections that targeted the superficial layers (I–IV) or the neighbouring regions of the upper lip of the sulcal cortex, namely, the VO-, the DLO- or the frontal association (FrA)-cortex, revealed scattered, diffuse terminal fields in the lateral hypothalamus (Fig. 4D). In the PAG, terminals were apparent not only after infections in the LO and VLO (Fig. 4A–D, H–J), but also after injections into the DLO-cortex (507-12; 552-12), although these were located more dorsally and were less strong than when the injection hit the LO-cortex.

In recent years, the Allen Institute has recorded data appertaining to random tracer-injections that have targeted the OFC (http://connectivity.brain-map.org/) (Oh et al. 2014). The data that are presented in the ABA are interesting also because gleaned from transgenic mice that had been genetically engineered to permit targeting of specific cell types. As soon as ABA-images appertaining to injections into the OFC were published, these were evaluated in parallel with the data that arose from our own injection experiments (Suppl. Table 1). The outcomes of our own injections corresponded well with the data that are recorded in the ABA. However, the mapping of data in the ABA ends at the level of the spine–medullary junction, whereas axons projecting from the OFC continue distally, terminating in the lateral horn of the spinal cord (Fig. 7C).

### The LO/VLO-cortex-derived projection in general

The projection from the LO/VLO-cortex extended as a straight, compact bundle of descending axons directed towards the hypothalamus (Fig. 7A). It has a length of 7.5 mm in rats and one of 4.5 mm in mice. From the injection site, it coursed through the caudatoputamen and the internal capsule to the level of the posterior lateral hypothalamus (Figs. 3G, 7). The angle between the axonal bundle and the upper surface of the brain is 55°; that between the bundle and the medial surface of the brain is 12° (as revealed on 3D-images in the ABA; Fig. 7A, B). From the posterolateral hypothalamus, two separate bundles left the cerebral peduncle coursing in an upward direction at an angle of 95° towards the rostral PAG, and at an angle of 115  towards the distal PAG (Fig. 7A). A third bundle left the cerebral peduncle caudally, one branch extending to the medial pontine nuclei (Pn) and a second to the reticulotegmental nucleus (RtTg) of the pons (Fig. 7B). A third branch continued distally in the cerebral peduncle and distributed axons bilaterally at various levels of the brainstem, with contralateral targets predominating (Fig. 7A, C). In cross sections of the brain, the fan-shaped axons were distributed perpendicular to the surface of the brain and gave rise to rich terminal fields in all parts of the reticular formation in the pons and the medulla oblongata (schematic in Fig. 7C). The terminal fields were most patent after immunostaining of the transported GFP-tracer with antibodies that were directed against GFP (222-13, 250-13, 556-12).

The projections from the LO- and the VLO-cortices were always bilateral, but were more abundant ipsilaterally until the end of the midbrain (Fig. 7C), with one exception: the projection from the VLO-cortex to the dorsal parabrachial leaflet (LPBreL) was more apparent contralaterally than ipsilaterally (Figs. 4j, 7C). In the pons and the medulla the contralateral projections were always stronger, except in the reticulotegmental (RtTg), and the pontine (Pn) nuclei. Caudal to the decussation of the pyramidal tract, they were more pronounced on the ipsilateral side of the spinal cord (Fig. 7C).

After injections into the OFC of *Pvalb*-Cre- (Fig. 4G) or *VGAT*-Cre mice (not shown), terminals were revealed at the level of the thalamus and that of the parvafox nucleus (Fig. 4G) in the hypothalamus, but only rarely more distally in either the PAG or the brainstem.

The range of possible targets of the LO- and the VLO-cortices varied somewhat: when the injections hit primarily the VLO-cortex, the PV2 and the LDTg in particular were strongly stained, whereas the parvafox- and the Su3 nuclei received fewer terminals. The primary and the secondary visual cortices were also stained, as was the perirhinal area; likewise the parabrachial (LPBreL), but not the basolateral nucleus of the amygdala (BLA). The bundles of axons were likewise located more medially in the caudatoputamen and the terminals in the Su3 nucleus were restricted dorsomedially, over the oculomotor nucleus. When the injection hit the LO-cortex, the density of labelled terminals was greater in the parvafox and Su3-nuclei, and endings were also observed in the basolateral nucleus of the amygdala (BLA), the ethmoid thalamic nucleus (Eth) and the mediodorsal thalamic nucleus (MDC).

When tracers were injected into the prelimbic cortex (PrL), terminals were widespread in the lateral hypothalamus, but were rare in the region of the parvafox nucleus (Sesack et al. 1989; Takagishi and Chiba 1991; Vertes 2004). They were notably absent from the region that was occupied by the parv-immunoreactive neurons in the parvafox nucleus, as revealed by immunofluorescence with another fluorochrome on the same section (Injection 130-13, not shown).

Injections into the insula (AIV and AID) (e.g. 507-12), gave rise to diffuse terminal fields in the parvafox nucleus but endings targeted the PAG, albeit in other quadrants.

To attain the superior lip of the rhinal fissure, where the orbital cortex is located, the injection needle traversed cortical areas on the dorsal (FrA, M2) and the dorsolateral (M1) aspects of the prefrontal cortex. Leakage of the tracer along the needle track sometimes involved these dorsal cortical areas in the injection. When area M2 was involved, it may have contributed to some of the terminal labelling seen in the brainstem. However, area FrA does not appear to project substantially to the hypothalamus or brainstem, because in two experiments in which the injection was accidentally made into area FrA by itself (553-12; 83-13), only a few terminals were observed in the hypothalamus or PAG.

### Detailed course of the axons and the location of their terminal fields

The description is limited to the regions in which novel projections arising from the VLO/LO cortex were observed (e.g. parvafox, Su3 and PV2). Observations that confirm already known terminal fields of the LO/VLO cortex—respectively, differences between already known projections of the LO-VLO cortex—are reported in the schematic drawing of Fig. 7C.

#### Hypothalamic targets of the OFC

The axonal bundles from the CPu merged to form the medial-most tip of the internal capsule at the level of the tuberal hypothalamus (Fig. 4A–D, G). Ventromedially, fine collaterals detached like a “pony-tail” from these axons (Figs. 3G, inset, 4A, C) and proceeded ventromedially to the region that is occupied by the parvafox nucleus (Figs. 3G, 4A–G). These fine axons branched profusely and terminated as synaptic “boutons” in an horizontal column—with a length and a breadth of 1 mm and 0.5 µm, respectively, in rats, and of 0.5 µm and 0.2 µm, respectively, in mice—which was sandwiched between the optic tract and the fornix. The fine axons sent out collaterals along their entire hypothalamic course. They were oriented perpendicular to the parental shafts and often ran in parallel with the dendrites of the Parv-expressing neurons (Celio et al. 2013). Most Parv- and Foxb1-expressing neurons in the parvafox nucleus received a substantial input from a few collaterals via repetitive axo-dendritic (and axo-somatic) synapses, and also sampled activity via non-repetitive *boutons en passant*. That the terminals from the OFC impinged on neurons in the parvafox nucleus was confirmed by injecting a Cre-dependent AAV-tomato tracer into its Parv and Foxb1-expressing neurons. The OFC-derived terminal endings abutted on the surfaces of the cell bodies and the dendrites, which were revealed with the tomato tracer (Fig. 4E, F). Hence, the presence of synaptic contacts is conceivable, and has been definitively proved by electron microscopy (see suppl. Figure 1).

The coarse parental axons of the cerebral peduncle curved upwards dorsomedially to form the periventricular system. A slender bundle of axons arising from the cerebral peduncle traversed the most distal portion of the parvafox nucleus (bregma level: − 2.2) and innervated the Gemini nuclei (Gem), which are located dorsomedially (Fig. 6E). The terminal field innervating the Gemini nuclei (ipsilateral > than contralateral) had a larger diameter than the one depicted in a standard mouse brain atlas (Paxinos and Franklin 2013).

#### Mesencephalon

At a slightly more distal level, namely, at the location in which the substantia nigra appears in the midbrain (bregma level: − 2.7), another bundle of coarse axons emitted from the cerebral peduncle in the same dorsomedial direction heavily innervated an oval volume that occupied a large portion of the ventral tegmental area (VTA) (Fig. 6F). It also gave rise to terminals in the PBP, the PN and PiF (Fig. 6F). This pattern of endings was observed after injections into both the LO- and the VLO-cortices, but the staining was more intense in the former than in the latter case. A projection from the LO-cortex to the VTA-region has indeed been reported in a trans-synaptic retrograde study using rabies viruses as a tracer (Ogawa et al. 2014; Watabe-Uchida et al. 2012).

**Fig. 5:**
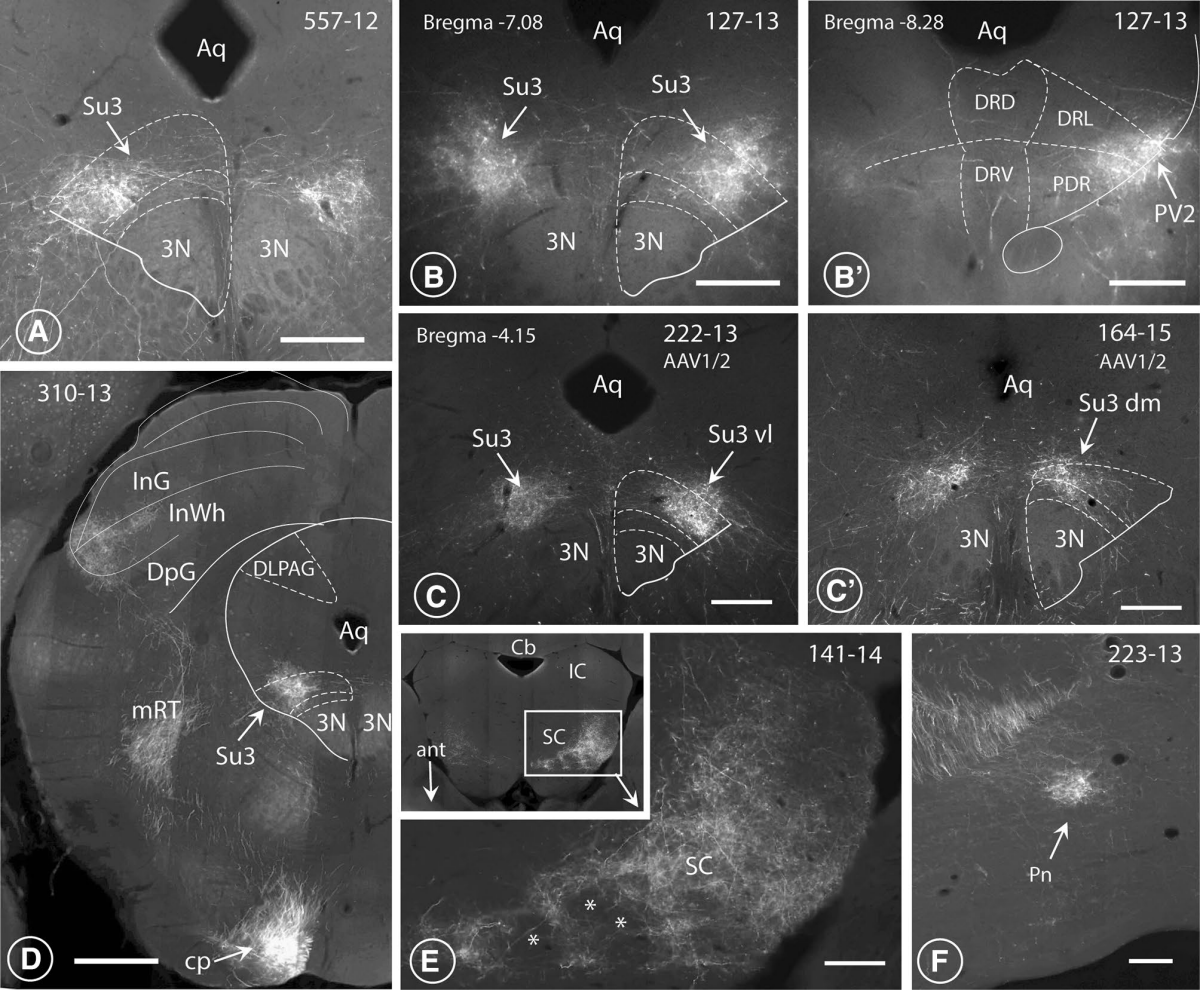
Terminal endings in the midbrain. Fluorescence images of LO-cortex-derived terminals in the Su3- and the PV2 nuclei of the PAG. **A, B, B′**′ Endings of the axonal collaterals of OFC-derived projections in two rats (specimen 557-12 and 127-13, injections depicted in Fig. 3f, g), which form a cap over the ventrolateral part of the oculomotor nucleus (3N; **A, B** compare also Fig. 3m) referred to as the Su3 nucleus (Carrive and Paxinos 1994). Slightly distally, endings terminate in the PV2 nucleus (**B**′ compare also with Fig. 4i). *Aq* aqueduct, *DRD, DRV, DRL* dorsal, ventral and lateral parts of the dorsal raphe nucleus, *PDR* posterodorsal raphe nucleus. Scale bars: **A** 0.2 mm. **C** After the injection of a tracer into the LO-cortex of a mouse (specimen 222-13, depicted in Fig. 3a), labelled terminals were observed ventro-laterally in the Su3 nucleus (Su3 vl, arrow). Scale bar: 0.2 mm. **C**′ Injecting the tracer in the VLO-cortex (specimen 164-15, depicted in Fig. 3d) revealed the presence of terminals in the dorsomedial part of the Su3 (Su3 dm, arrow). Scale bar 0.2 mm. **D** In addition to the Su3-region, LO-derived axons ascend from the cerebral peduncle to terminate in the most lateral edge of the intermediate grey (InG) and white layers (InWh) of the superior colliculus (SC). Terminals from the VLO-cortex are located more dorso-medially. *mRT* mesencephalic reticular formation, *DLPAG* dorsolateral PAG. Scale bar: 0.5 mm. **E** In horizontal sections, this terminal field in the superior colliculus (SC) had a honeycomb- (*) or “fence”- like appearance (141-14), see also Beckstead (1979). *Ant* anterior, *Cb* cerebellum, *IC* inferior colliculus, *SC* superior colliculus. Scale bar: 0.15 mm. **F** Terminals were observed also in the medial pontine nuclei (Pn) and in the reticulotegmental nucleus (RtTg; Figs. 6f, 7b, c). Scale bar: 0.1 mm

Axons of the cerebral peduncle subdivided into two formations. One was located medially and extended to the mid-portion of the PAG (Fig. 7A, C). It sent out collaterals, which ended in a column of terminals in the ventrolateral part of the Su3 nucleus (Fig. 5A–C), as has been indicated by others (Beckstead 1979). This terminal field corresponded in location to the terminal field of axons deriving from both the Parv as well as the Foxb1-subpopulation of neurons in the parvafox nucleus of the hypothalamus (Bilella et al. 2016; Celio et al. 2013). Injections preponderantly in the VLO-cortex lead to terminals in the dorsolateral part of the Su3 (Fig. 5C′) and additionally to endings located in the dorsolateral PAG (not shown). The second termination field was located slightly more caudally, in the region of the PV2 nucleus (Figs. 4i, 5B′) (Celio et al. 2013). It extended to the laterodorsal tegmental nucleus (LDtg) of the PAG (Fig. 7A, C), as has been already reported (Leichnetz et al. 1987b). The existence of connections between the OFC and the oculomotor region was confirmed earlier by retrograde tracing techniques (Leichnetz and Gonzalo-Ruiz 1987a; Leichnetz et al. 1987a).

**Fig. 6:**
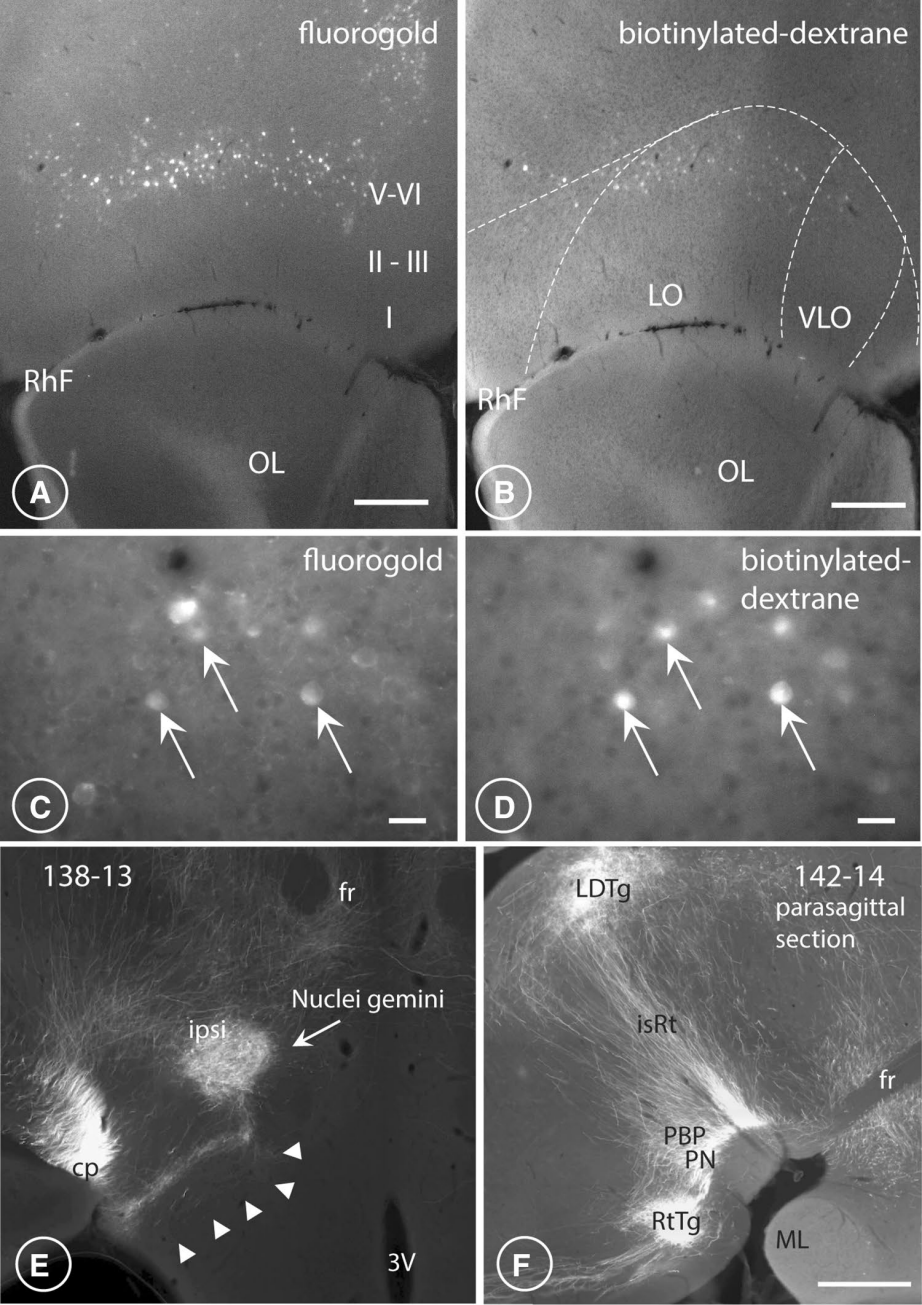
LO/VLO pyramidal neurons send collaterals to both hypothalamus and PAG. Terminals in the Gemini (Gem) and reticulotegmental nucleus (RtTg). Axons terminating in the parvafox nucleus are the collaterals of fibres that continue their course distally to innervate the ventrolateral PAG. Transverse sections through the rat OFC after fluorogold (**A, C**) and BDA (**B, D**), had been simultaneously injected into the ventrolateral region of the hypothalamus that harbours the parvafox nucleus (fluorogold) and into the ventrolateral region of the PAG in which the Su3- and the PV2 nuclei reside (BDA). Pyramidal cells in layers V–VI of the LO/VLO-cortex were double-labelled (white arrows in **C, D**) with two different retrogradely transported dyes. Fluorogold injected in the ventrolateral hypothalamus revealed the presence of a larger number of labelled neurons in the orbital and the medial portions of the prefrontal cortex than did BDA injected in the ventrolateral PAG. *OL* olfactory lobe, *RhF* rhinal fissure, *I–VI* cortical layers. Scale bars: **A, B** 0.3 mm; **C, D** 0.03 mm. **E** The ipsilateral nucleus Gemini (Gem), receives a strong innervation, particularly from the LO-cortex, visible also on the contralateral side. Notice the tendril-like bundle of axons (arrowheads) joining the nucleus Gemini from the ventromedial part of the cerebral peduncle. Scale bar 0.5 mm. **F** In this parasagittal section (rostral to the right), in addition to PBP and PN, also the rich innervation of the reticulotegmental nucleus (RtTg) is visible. A large number of axons perpendicular to the brain surface course in the isthmic reticular formation (isRt) to innervate the laterodorsal tegmental nucleus (LDTg). Scale bar: 0.5 mm

**Fig. 7:**
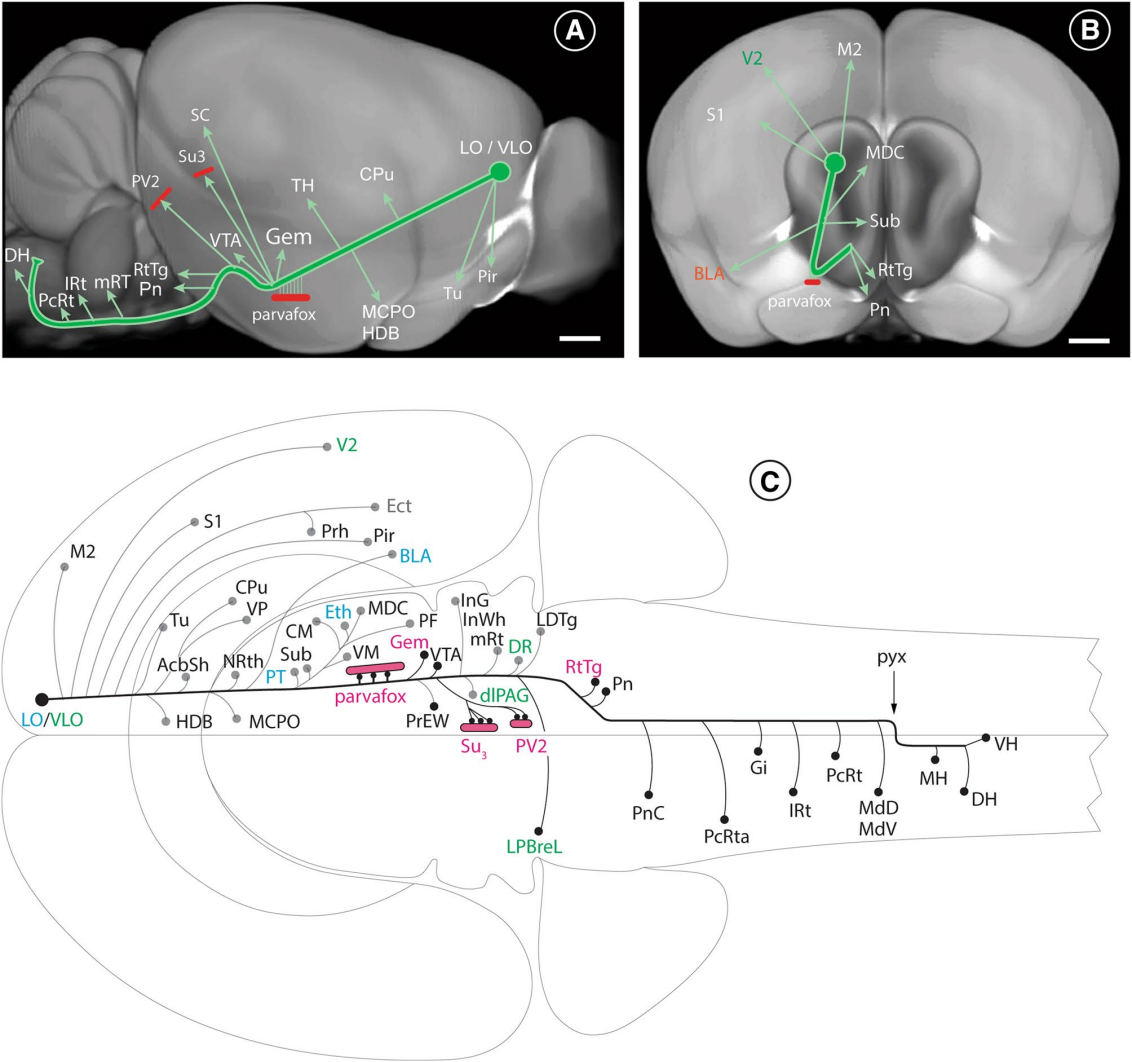
Schematic representation of the projections from the LO/VLO cortex to the rest of the brain. The courses of the major axonal branches deriving from the LO-cortex in mice are here superimposed to reconstructions from the Allen Brain Atlas (experiment no. 112306316). **A** In this lateral view, the bulk of the axons descend from the LO-cortex to the lateral hypothalamus at an angle of ~ 120° relative to the surface of the brain (see also Fig. 3e). During their course, axons send out branches to various parts of the brain: to the olfactory tubercle (Tu), to the piriform cortex (Pir), the caudate–putamen (Cpu), various nuclei of the thalamus (TH), the nucleus of the horizontal limb of the diagonal band (HDB), the magnocellular hypothalamic nucleus (MCPO), the parvafox nucleus, the Gemini nucleus (Gem), the ventral tegmental area (VTA), the superior colliculus (SC), the Su3 nucleus and the PV2/LTDg-region. The main track of parent axons arches and sends out projections to the pontine nuclei (Pn) and the reticulotegmental nucleus (RtTg). It continues its course more distally to innervate the entire dorsal part of the reticular substance PcRt, IRt, mRT, MdD, MdV, and ends in the lateral horn of the cervical spinal cord. **B** In this frontal view the LO-cortex-derived projection follows a low-angled course relative to the sagittal plane. It sends out branches to the motoric and the sensory cortices, the basolateral nucleus of the amygdala (BLA), the thalamus [central mediodorsal (MDC) and submedius-(Sub)], the pontine nuclei (Pn) and the reticulotegmental nucleus (RtTg). **C** Flat view of the projections from the LO- and the VLO-cortices to various parts of the brain, as represented in Swanson’s atlas (Swanson 2004). The axons deriving from the LO/VLO cortices send out branches and collaterals at various levels, mainly ipsilaterally, until the end of the midbrain. In the medulla oblongata, the thicker collaterals project contralaterally and the thinner ones ipsilaterally (to simplify the drawing, the collaterals are drawn only on the side of the brain where they are more pronounced). The axons cross to the contralateral side via the pyramidal decussation (pyx). Many of the OFC projections to individual targets depicted here, have been described by earlier authors: we additionally observed substantial innervation of the parvafox nucleus in the ventrolateral hypothalamus and of the Su3- and the PV2 nuclei in the ventrolateral PAG. Furthermore, we recognized terminals in the Gemini nucleus (Gem), in the lateral parabrachial nucleus LPBreL (all indicated in red) and in the reticular formation. We could also distinguish between projections that emanated from either the VLO- (green) or the LO-cortex (light-blue). Each of the indicated structures is mentioned in the list of abbreviations. *LH* lateral horn, *DH* dorsal horn, *SC* superior colliculus, *VH* ventral horn of the spinal cord. Scale bars in **A, B**: 0.6 mm

A lateral formation of fibres dissociated in the *Crus cerebri* from the one that was destined to innervate the region of the Su3-nucleus. These fibres projected to, and terminated in the *tectum* of the midbrain, specifically, in the lateral edge of the intermediate grey (InG) and white (InWh) layers of the superior colliculus, where they formed a spherical (223-13, LO-Injection) or a honeycomb or “fence”-like structure (Figs. 5D, E, 7A, C), as has been before described (Beckstead 1979). These terminals were identified both in LO- as well as in VLO-cortex injected specimen. Those deriving from the VLO-cortex were located more dorso-medially in respect to those from the LO-cortex. Terminals were observed also in the medial pontine nuclei (Fig. 5F) and also in the reticulotegmental nucleus (Fig. 7).

#### Metencephalon

The OFC-derived terminals around the neurons in the PV2 nucleus continued distally to the dorsal portion of the LDTg and to the sublaterodorsal nucleus. A slender comma-shaped field of terminals was evident contralateral to the injection site in the region of the lateral parabrachial nucleus (LPB) (Figs. 4J, 7C), which is sandwiched between the LPB and the MPB. This region may be occupied by one of the CCK-expressing group of neurons that relay thermosensitive information to the lateral hypothalamus (Geerling et al. 2016). This projection was observed only when the injections involved the VLO-cortex.

The parental axons continued caudally to the cerebral peduncle of the pons, wherefrom they spread out in a fan-like manner to both of its halves (Fig. 7C). After the distance of a few micrometres in the dorsal direction, the axons branched into collaterals of unequal thickness. The thicker ones innervated the contralateral reticular formation (Gi, IRt and PcRt). The terminal fields were confined by the sharp boundaries of the prepositus hypoglossi nucleus upwards, by those of the spinal trigeminal nucleus sidewards and by those of the lateral paragigantocellular nucleus downwards. A field of higher-density terminals was observed just laterodorsal to the compact portion of the nucleus ambiguus (Fig. 7C) in the parvicellular reticular nucleus (PcRtA). On the ipsilateral side, the field of projection of the OFC-derived axons and that of the terminals were identical to those on the contralateral one, but were less dense and less intensely stained.

#### Medulla oblongata

Rostrally, the contralateral IRT and the GIA received most of the terminals, and the Amb none (Fig. 7C). More distally, the upper portions of the contralateral IRt, MdV and MdD, received terminals from the LO-cortex. Ipsilaterally, the density of the terminals was much lower.

### Cre-dependent tracers

Injections of Cre-dependent tracers into the OFC of Parv-Cre mice lead to the impregnation of neurons in various layers (Fig. 3J, K). A small contingent of axons left the cortex above the external capsule, passing through the caudatoputamen and occupying the internal capsule. Their position therein corresponded to that of the projections of the pyramidal neurons in layers V–VI, and they distributed terminals to the same sites [whole extent of the parvafox (Fig. 4G)], Su3 nucleus, PV2 nucleus (a few). In the thalamus, the endings were detected in the mediodorsal central nucleus (306-15; 394-14), in the submediodorsal and the ventral nuclei (309-15, 394-14) and in the Gemini nuclei (394-14). They were also observed in the laterodorsal tegmental nucleus (LDTg) and in the reticulotegmental nucleus (RtTG; 307-15) of the brainstem; they ended in the central grey-alpha region of the pons.

### Retrograde tracing experiments

Concomitant retrograde tracing with fluorogold from the ventrolateral region of the hypothalamus that harboured the parvafox nucleus and with BDA (MW: 3000) from the ventrolateral PAG revealed layer-V pyramidal cells in the OFC to contain both tags (Fig. 6A–D). Hence, collaterals emanating from the axons of some of the pyramidal cells in layer V–VI innervated the parvafox nucleus whilst the main fibres continued distally to innervate the PAG. Similar findings have been documented for neurons of the VLO- and the LO-cortices, which collateralize first to the caudatoputamen and then to the core of the accumbens (Reynolds and Zahm 2005).

Retrograde, trans-synaptic labelling with Cre-dependent rabies-virus constructs, which were injected into the region that was occupied by the parvafox nucleus in *Pvalb*-Cre/*Foxb1*-Cre mice, confirmed the presence of tagged neurons in the LO- and the VLO-cortices (Fig. 3L) but also in the medial prefrontal cortex (MO). The MO-neurons probably label single Foxb1-expressing neurons located in the region surrounding the parvafox. In the *Pvalb*-Cre mice, the label was observed in layers V–VI of the LO-cortex, at the location of the upper lip of the rhinal sulcus. In the *Foxb1-*Cre mice, the label was detected also in the adjoining VLO- and the VO-cortices. This pattern of distribution indicates that all these OFC regions target the Foxb1-expressing sub-population of the parvafox nucleus.

### Relationship between terminals in the PAG

At the level of the PAG, the relationship between endings deriving from neurons in the LO-VLO-cortices and those emanating from Parv- or Foxb1-expressing neurons were studied in the confocal scanning microscope after a double injection of fluorescence tracers. Axons from the OFC were revealed with a Cre-independent EGF-tracer and those in the parvafox nucleus with a Cre-dependent tomato tracer (Fig. 3M). Although most of the terminals impinged on resident neurons in the PAG, contacts between the two populations of terminals were common (not shown). Terminals deriving from the parvafox nucleus were sometimes pre-synaptic and sometimes post-synaptic relative to those that emanated from the LO-cortex.

### Ultrastructure

Axons from the OFC ended primarily with asymmetric synapses, characterized by round synaptic vesicles in the pre-terminal endings and a thick postsynaptic density (suppl. Figure 1A). In the region of the parvafox, axons of the OFC themselves received both excitatory (suppl. Figure 1B) as well as inhibitory, symmetric synapses, characterized by oval synaptic vesicles (suppl. Figure 1C).

## Discussion

The hypothalamic target of the murine LO/VLO cortical regions—corresponding to the Area 13a and 13 m/l in primates—is the newly described parvafox nucleus and its targets Su3 and PV2 in the PAG.

By drawing on self-replicating virus-based tracing tools, which enhance the sensitivity of connectivity studies, we have defined three targets of the LO/VLO cortices that have been hitherto overlooked, namely, the parvafox nucleus which is located in the ventrolateral hypothalamus, and the Su3- and the PV2 nuclei which are serially located ventral to the aqueduct in the periaqueductal grey matter (PAG).

Both LO and VLO innervate these three targets, although partly in different portions: in the SU3-nucleus, for example, the VLO-projection is located more medially than the one arising in the LO-cortex. This medial location of the VLO-projection is also observed in other part of the brain like the caudatoputamen, and superior colliculi. In the ventral tegmental area, the projection from the LO-cortex is more important than the one deriving from the VLO-cortex. There are also specific projections for each one of these two subdivisions of the OFC. The VLO-cortex for example, innervates parts of the visual cortex (Reep et al. 1996), the dorsal raphe (DR), the dorsolateral column of the PAG, a region that is also target of the Foxb1-subpopulation of neurons of the parvafox nucleus (Bilella et al. 2016) and has a specific projection to the contralateral, dorsal parabrachial nucleus (LPBrel). The LO-cortex innervates the ethmoid thalamic nucleus, the basolateral amygdala (Groenewegen et al. 1990; McDonald et al. 1996) and the ventral part of the submedius nucleus (Craig et al. 1982). In addition, terminals of the LO/LO cortex were also observed in nuclei not mentioned in the previous literature, namely the Gemini nuclei, the lateral parabrachial nucleus, the pontine nuclei, the reticulotegmental nucleus, the reticular substance of the brainstem and the lateral horn of the spinal cord. No major differences in the projection patterns were detected between rats and mice and in both species the projections to parvafox, Su3 and PV2 were of comparable intensity.

Using a Cre-dependent rabies virus (Wall et al. 2010; Wickersham et al. 2007b) the afferences to the Parv- and the Foxb1-expressing neurons could be studied separately and selectively in the corresponding Cre-mice. The input to the Parv-expressing neurons that represent the core of the parvafox nucleus originates mainly in the LO-cortex, whereas the inputs to the Foxb1-expressing neurons derive from the medial (IL, PrL), the orbitofrontal (MO, VO, VLO, LO) and even the lateral prefrontal cortices. These projection patterns correspond well with those that have been revealed by retrograde tracing with peroxidase from the rat ventrolateral hypothalamus [(Allen and Cechetto 1993) their Fig. 5a].

Our study is the second of its kind in which the anterograde projections of the LO-VLO cortex throughout the entire brain have been mapped. In the other investigation, which appeared four decades ago, autoradiographic techniques were implemented to map the staining profiles of OFC-derived projections after the injection of tritiated amino acids (Beckstead 1979). In parallel to our study, a large body of data appertaining to stereotactic injections into the OFC have been published during the past 3 years in the ABA (http://www.brain-map.org), some of which reveal patterns of labelling of the terminal fields that are almost identical to those that we observed. Our data and those that are recorded in the ABA largely confirm the previous, precise observations of Leonard (1969), Beckstead (1979) and Reep et al. (1996) in the rat as well as for VLO in the cat (Craig et al. 1982). To the list of known targets, we append three chemically defined sites (the parvafox-, the Su3- and the PV2 nuclei). Our knowledge of its existence (Bilella et al. 2016; Celio et al. 2013; Meszar et al. 2012), guided our attention to recognize the parvafox, a small elongated neuronal aggregates as targets of the projections of the LO- and the VLO-cortices [“C*hance favours the prepared mind*” (citation of a statement by Louis Pasteur)]. In the aforementioned autoradiographic study, the presence of terminals in a tiny oval region that lay along the optic tract was actually documented pictorially [injection 8, Fig. 6 (Beckstead 1979)], but not mentioned in the text. From its position, we presume it to be the parvafox nucleus. The cortical projection proceeded caudally to the ventrolateral PAG, namely, to a region that lay dorsal to the nucleus of the third nerve, which was referred to by Beckstead as a “subaqueductal portion”, and which approximately corresponds to the terminal field that we observed in the supraoculomotor region (the Su3 nucleus). A terminal field at precisely the same location has been described in feline brains after the injection of tracers into the VLOβ-region [Fig. 15 in Craig et al. (1982)]. Beckstead observed the projection to terminate in the laterodorsal tegmental nucleus, probably in the region that we refer to as the PV2 nucleus, owing to its composition of parvalbumin-expressing neurons (Celio et al. 2013). In the publication by Jasmin (Jasmin et al. 2004), tracers were injected into a region which, according to Price’s group is probably still the LO-cortex but which the former authors defined as the AIV [images 1a and b in Jasmin et al. (2004)]. The distribution of the terminal fields accorded with our own observations, although those in the lateral hypothalamic parvafox nucleus were more sparse (their Fig. 3F). The terminals that were detected in the “ventrolateral wings of the dorsal raphe nucleus” probably correspond to those in the region of the Su3-region (their Fig. 3G).

Using the mouse connectivity programme of the Allen Database (ABA; http://www.brain-map.org) and entering as source structures various areas of the prefrontal cortex without any filter for mouse line or tracer type, the results corresponded well with our own data. For the purpose of our study, the most relevant injections in the ABA were the numbers 112423392 and 112306316, since they were performed in mice of the same wild-type strain (C57/BI6). But also injections into the genetically modified lines A930038C07Rik-Tg1-Cr (Cre-recombinase confined to layer V; no. 168164972) and Rbp4-Cre_KL100 (Cre-recombinase confined to layer V; no. 287769286) revealed patterns of projections that accorded with the observations in our own.

The precise targets of the OFC-derived projection in the PAG are, with a high degree of axial and radial specificity, the Su3 nucleus, which is located slightly dorsolateral to the oculomotor nucleus, and the parvalbumin-expressing ones in the PV2 nucleus, which is located in the posterodorsal raphe nucleus (PDR) and rostral portion of the dorsolateral tegmental nucleus (LDTg). With a view to mapping the frontal eye field, Leichnetz (Leichnetz et al. 1987b) injected retrograde tracers into the oculomotor nucleus, to which end, the needle was oriented slightly obliquely (their experiments OMR2 and OMR3), thereby perforating the supraoculomotor region (harbouring the Su3 nucleus). Retrogradely labelled neurons occurred primarily in the cortex of the dorsomedial shoulder (the putative rodent homologue of the primate frontal eye field), scatterings of stained cells were nevertheless observed throughout the entire OFC (Leichnetz et al. 1987b).

The Su3 nucleus is known to project to the contralateral rostral- and the caudal ventrolateral medulla, which together constitute the sympathetic cardiovascular control centre (Chen and Aston-Jones 1996; Van Bockstaele et al. 1989). The Su3 nucleus receives inputs from the medial cerebellar nucleus (fastigium) (Gonzalo-Ruiz and Leichnetz 1987; Gonzalo-Ruiz et al. 1990) and from the parvafox nucleus (Bilella et al. 2016; Celio et al. 2013) and is activated during predation [of insects as well as by cats (Comoli et al. 2012)]. The connections and functions of the PV2-nucleus are yet unknown.

The hypothalamus and the PAG, two regions of the brain that harbour the parvafox-, and the Su3- and the PV2 nuclei, are themselves interconnected. This is a general organization, which has been described for the lateral hypothalamus broadly and for the ventrolateral PAG in particular (Floyd et al. 2000, 2001). The parvafox nucleus, does not reciprocate the projections from the OFC (Bilella et al. 2016; Celio et al. 2013), either directly or indirectly, via an innervation of the mediodorsal thalamic nucleus. The findings of the double retrograde tracing experiments that were conducted by Gabbott and his colleagues (Gabbott et al. 2005), as well as by ourselves, indicate that the projection from the LO/VLO-orbitofrontal cortex is—at least in part—serial, with the same axon successively innervating multiple subcortical targets via collaterals.

Part of the projection from the LO/VLO-cortex stems from parvalbumin-expressing neurons, which represent a sub-population of cortical GABAergic cells (Celio 1986). Long-range-projecting neurons expressing NO-synthase have been observed to connect cortical areas (Tamamaki and Tomioka 2010) and parvalbumin-expressing GABAergic ones are known to project from the medial prefrontal cortex to the nucleus accumbens (Lee et al. 2014). These parvalbumin-expressing neurons in the OFC are well positioned for a “top-down” inhibitory control of subcortical processes (Fuster 2008). In our study, their GABAergic nature was suggested by the presence of GAD-immunoreactive axonal endings on neurons in the parvafox nucleus. However, injections of Cre-dependent tracers into *VGAT*-ires-cre mice (in which Cre-recombinase is expressed in the bodies of GABAergic neurons), revealed the presence of only a few projections outside the cortex (not shown). In the ABA, the injection of a tracer into the VLO of a *Slc32a*1-IRES-Cre mouse (VGAT) revealed no projecting axons (injection no. 309580102).

The rat LO-cortex has been hitherto regarded as a constituent of the orbital network (Krettek and Price 1977) and to be more of a “sensory” than a visceromotor region. In addition to many other cerebral sites that have been reported by various authors, we have demonstrated the LO-cortex to project to circumscribed horizontal columns of neurons in the parvafox nucleus of the ventrolateral hypothalamus in the SU3 and the PV2 nuclei of the PAG. These findings suggest that the subdivision of the OCF-cortex into sensory and visceromotoric regions may not be as absolute in rodents as it is in monkeys (Price 2007).

The lateral hypothalamic region in which the parvafox nucleus is located receives inputs from various olfactory regions and from the amygdala (Price et al. 1991). Each of these areas has reciprocal connections with the OFC, which the findings of our study have revealed to target, non-reciprocally, the neurons of the parvafox nucleus. Furthermore, the olfactory tubercle projects to the Gemini nuclei, which are also targeted by the LO/VLO-cortex-derived projection and by the parvafox. It remains to be established whether the parvafox- or the Gemini nuclei have any distinct olfactory functions. The olfactory projection to the ventrolateral hypothalamus may regulate autonomic or neuroendocrine functions or related behaviours (Price et al. 1991), or it may simply contribute olfactory information to be integrated with other influences.

In addition to its involvement in the processing of olfactory and gustatory information, the OFC also controls the cardiovascular and the respiratory systems (Fuster 2008). The pioneering work of electrophysiologists revealed the most prominent consequences of stimulating Area 13 of the OFC in primates to be manifested in the cardiovascular and the respiratory systems (Fuster 2008). The effects include changes in blood pressure, heart rate, cardiac dynamics, respiratory rate and skin temperature (Bailey and Sweet 1940; Chapman et al. 1948; Delgado et al. 1960; Hall and Cornish 1977; Kaada et al. 1953; Sachs et al. 1949; Spencer 1894), which can even lead to cardiac histopathology (Hall and Cornish 1977). An exploration of various parts of the brain with electrodes whilst stimulating the OFC with strychnine (neuronography) has permitted a mapping of the connections of Area 13 with the lateral hypothalamus, particularly with the ventrolateral region in which the median forebrain bundle resides (Sachs et al. 1949; Ward and McCulloch 1947). Interestingly, some of the autonomic effects that are evoked by stimulation of the OFC may be likewise elicited by stimulating the lateral hypothalamic region (where the parvafox is located) and the ventrolateral PAG (where the Su3 and PV2 nuclei are found), which receive these cortical afferences. (Allen and Cechetto 1992, 1993; Bailey and Sweet 1940; Chapman et al. 1948; Delgado et al. 1960; Fernandez De Molina and; Hunsperger 1962, 1956; Gelsema et al. 1989; Hall and Cornish 1977; Hess 1957; Kaada et al. 1953; Loewy 1991; Ruggiero et al. 1987; Sachs et al. 1949; Verberne 1996; Verberne and Owens 1998; Yasui et al. 1991).

In addition to its role in olfactory processing and in visceromotor activity, the OFC is best known for its involvement in the expression of emotion and in reward-driven decision-making (Bechara et al. 2000; Rolls 2000; Schultz et al. 2000). This co-habitation of sensory, autonomic and behavioural networks in the OFC permits the “integration of primitive autonomic mechanisms (such as are associated with instinctive urges or emotional reactions) with neural activities at the highest functional level of the brain” (Clark Le Gros and Meyer 1950).

Patients with lesions in the OFC [specifically of the VM], manifest an impaired ability to generate anticipatory “skin conductance responses” (SCR) to a conceived outcome of an action (Bechara et al. 2005; Damasio 1996). SCRs are emotional signals (somatic marker) that are generated by the activity of the autonomic nervous system, of which the hypothalamus is the main organizer (Hess 1947, 1957).

Future studies will reveal whether the OFC → parvafox → PAG network that we have delineated in our study is the scaffold on which the first-named region engages the autonomic nervous system. In analogy to the SCRs, the OFC → parvafox → PAG network could also affect the physiology of the cardiovascular and the respiratory systems (Rainville et al. 2006) and be involved in pathologies thereof that relate to disturbances in high mental activities (Pickering et al. 1996).

## Electronic supplementary material

Below is the link to the electronic supplementary material.

Supplementary material 1 (DOCX 17 KB)

Supplementary material 2 (TIF 1894 KB)

Supplementary material 3 (TIF 12517 KB)

Supplementary material 4 (DOCX 55 KB)

## Abbreviations

3N: Oculomotor nucleus
3PC: Parvicellular part oculomotor nucleus
AAV: Adeno associated virus
AcbSh: Accumbens nucleus shell
AID: Agranular insular cortex, dorsal part
AIP: Agranular insular cortex, posterior part
AIV: Agranular insular cortex, ventral part
Amb: Ambiguus nucleus
BDA: Biotinylated dextrane
BLA: Basolateral amygdaloid nucleus anterior part
Cb: Cerebellum
Cl: Claustrum
CM: Central thalamic nucleus
cpd: Cerebral peduncle
CPu: Cudatoputamen
DA8: Dopamine cells
DH: Dorsal horn spinal cord
DI: Dysgranular insular cortex
DLO: Dorsolateral orbital cortex
dlPAG: Dorsolateral PAG
DTG: Dorsal tegmental nucleus
DR: Dorsal raphe nucleus
Eth: Ethmoid thalamic nucleus
fmi: Forceps minor corpus callosum
FrA: Frontal association cortex
fv: Flat vesicles
Gem: Gemini hypothalamic nucleus
GFP: Green fluorescence protein
Gi: Gigantocellular reticular nucleus
GI: Granular insular cortex
GiA: Giggantocellular reticular nucleus alpha part
HDB: Nucleus of the horizontal limb of the diagonal band
IC: Inferior colliculus
IL: Infralimbic cortex
InG: Intermediate gray layer of the superior colliculus
InWh: Intermediate white layer of the superior colliculus
IRt: Intermediate reticular nucleus
isRT: Isthmic reticular formation
LDTg: Laterodorsal tegmental nucleus
LH: Lateral horn spinal cord
LO: Lateral orbital cortex
LPBreL: Lateral parabrachial nucleus
M1: Primary motor cortex
M2: Secondary motor cortex
MCPO: Magnocellular preoptic nucleus
MDC: Mediodorsal thalamic nucleus central part
MdD: Medullary reticular nucleus dorsal part
MdV: Medullary reticular nucleus ventral part
MO: Medial orbitofrontal cortex
mRt: Mesencephalic reticular formation
NRth: Reticular thalamic nucleus
OFC: Orbitofrontal cortex
OL: Olfactory lobe
PAG: Periaqueductal gray
parvafox: Parvafox nucleus
PBP (VTA): Parabrachial pigmented nucleus
PcRtA: Parvicellular reticular nucleus (anterior part)
PDR: Posterodorsal raphe nucleus
PF: Parafascicular nucleus
PIF (VTA): Parainterfascicular nucleus
Pir: Piriform cortex
PN (VTA): Paranigral nucleus of the VTA
Pn: Pontine nuclei
PrEW: Pre-Edinger Westphal nucleus
Prh: Perirhinal cortex
PrL: Prelimbic area
PT: Parataenial thalamic nucleus
PV2: PV2 nucleus (in LDTg)
py: Pyramidal tract
pyx: Pyramidal decussation
RFP: Red fluorescent protein
RhF: Rhinal fissure
rsv: Round synaptic vesicles
RtTg: Reticulotegmental nucleus
S1: Primary somatosensory cortex
SC: Superior colliculus
scp: Superior cerebellar peduncle
Su3: Supraoculomotor periaqueductal grey
Sub: Submedius thalamic nucleus
TH: Thalamus
TO: Optic tract
Tu: Olfactory tubercle
VH: Ventral horn spinal cord
VM: Ventromedial thalamic nucleus
V2: Secondary visual cortex
VLO: Ventrolateral orbitofrontal cortex
VM: Ventromedial prefrontal cortex
VO: Ventral orbitofrontal cortex
VP: Ventral pallidum
VTA: Ventral tegmental area
