# Supplementary material for: The orbitofrontal cortex projects to the parvafox nucleus of the ventrolateral hypothalamus and to its targets in the ventromedial periaqueductal grey matter
Source: Brain Struct Funct. 2018 Oct 12;224(1):293–314. doi: 10.1007/s00429-018-1771-5 (PMC6373537; doi:10.1007/s00429-018-1771-5)
Supplement: Supplementary file 1 — Supplementary material 1 (DOCX 17 KB) [file 429_2018_1771_MOESM1_ESM.docx]

Suppl, Table 1: Tracer injections into the LO- and the VLO-cortices that are represented in the Allen Database

| Injected Area | Exp. number | Genotype | Parvafox | Su3 | PV2 | Comments |
| --- | --- | --- | --- | --- | --- | --- |
| PL | 263106036 | [Rbp4-Cre_KL100](http://connectivity.brain-map.org/transgenic/search?search_type=line&search_term=Rbp4-Cre_KL100)  Enriched in layer 5 | avoided |  |  | Innervates a large area of the lateral hypothalamus |
| PL | 283019341 | [A930038C07Rik-Tg1-Cre](http://connectivity.brain-map.org/transgenic/search?search_type=line&search_term=A930038C07Rik-Tg1-Cre)  Expressed in layer 5 | Generally strong LHA |  |  | Innervates a large area of the lateral hypothalamus |
| ILA | 286313491 | [Rbp4-Cre_KL100](http://connectivity.brain-map.org/transgenic/search?search_type=line&search_term=Rbp4-Cre_KL100)  Enriched in layer 5 | + |  |  | Strong innervation of the lateral hypothalamus, minor in the region of the parvafox, More perifornical large terminal field. |
| **ORBvl (76%)** ORBl (8%)  ORBm  (2%) PL (9%), | 112423392 | C57BL/6J  WT | + | + dorsomedial | ++ | Injection slightly medial from the olfactory notch. |
| **ORBl (83%)**,  ORBvl (16%) | 112306316 | C57BL/6J  WT | ++ | ++  ventromedial | ++ | Injection that corresponds best to ours.  Gemini |
| **ORBl (65%),**  ORBvl (33%), Fr (2%)  , | 156741826  Olfactory notch | [Rbp4-Cre_KL100](http://connectivity.brain-map.org/transgenic/search?search_type=line&search_term=Rbp4-Cre_KL100)  enriched in cortical layer 5 | + | +  ventromedial | + | Faint in thalamus, nothing in hindbrain.  Gemini |
| ORBl, AiD, AiV | 180709230 | C57BL/6J  WT | (+) | + | (+) | Strong projection in the insular cortex  Gemini |
| **ORBvl**, **ORBl** MOS  ILA | 183618845  Olfactory notch | [Htr2a-Cre_KM207](http://connectivity.brain-map.org/transgenic/search?search_type=line&search_term=Htr2a-Cre_KM207)  Enriched in layer 5 and 6b | + | +  whole Su3 | + | Gemini |
| **ORBl (65%)**, ORBvl (35%) | 168164972  Olfactory notch | [A930038C07Rik-Tg1-Cre](http://connectivity.brain-map.org/transgenic/search?search_type=line&search_term=A930038C07Rik-Tg1-Cre) restricted to layer 5 | + | +  whole Su3 | ++ | Thalamus only in Sub, rest faint |
| ORBl, FRP, MOs, ORBvl, Ald, AOB | 288324211 | [Grp-Cre_KH288](http://connectivity.brain-map.org/transgenic/search?search_type=line&search_term=Grp-Cre_KH288)  GPR-Cre  Enriched in layer 2-3 | - | - | - | Reaches only until the striatum. Contingent of axons until brainstem |
| ORBl, FRP, MOs, ORBvl, Ald, AOB | 265713683 | [Gpr26-Cre_KO250](http://connectivity.brain-map.org/transgenic/search?search_type=line&search_term=Gpr26-Cre_KO250) Enriched in 5 and 6b | (+) | (+) | (+) | Faint |
| ORBl, Mop, ORBvl, Ald | 126353451 | [Syt6-Cre_KI148](http://connectivity.brain-map.org/transgenic/search?search_type=line&search_term=Syt6-Cre_KI148)  Enriched in layer 6a | (+) | (+) | (+) | Reaches thalamus but no terminals in the striatum. Few endings parvafox, Su3 and PV2 |
| ORBl, MOs, Ald | 159887627 | Nr5a1-Cre  Restricted to layer 4 | - | - | - | Reaches only striatum |
| ORBVl | 183471174 | Enriched in layers 2,3,4 | - | - | - | Reaches striatum, very few terminals in thalamus |
| ORBl, Mop, MOs, ORBvl | 278174451 | [Chrna2-Cre_OE25](http://connectivity.brain-map.org/transgenic/search?search_type=line&search_term=Chrna2-Cre_OE25) Enriched in layer 5 | (+) | (+) | (+) | Precise, small injection, slender projection |
| ORBl, Mop, MOs, ILA, ORBvl, Ald, Alv | 307766627 | [Etv1-CreERT2](http://connectivity.brain-map.org/transgenic/search?search_type=line&search_term=Etv1-CreERT2)  Enriched in layer 5 | (+) | - | (+) | No endings in striatum, only in thalamus, Gemini |
| ORBM, MOs, ACAd, PL, ILA, ORBvl | 496554237 | [Rbp4-Cre_KL100](http://connectivity.brain-map.org/transgenic/search?search_type=line&search_term=Rbp4-Cre_KL100)  Enriched in layer 5 | (+) | (+) | (+) | Terminals in the whole lateral hypothalamus, but not concentrated in the parvafox-nucleus. |
| ORBm, ILA, ORBvl | 265820216 | [Syt6-Cre_KI148](http://connectivity.brain-map.org/transgenic/search?search_type=line&search_term=Syt6-Cre_KI148)  Enriched in 6a | - | - | - | Reaches until thalamus. No terminals in striatum |
| ORBm, ACAd, PL, ILA, ORBvl | 277956496 | [Htr2a-Cre_KM207](http://connectivity.brain-map.org/transgenic/search?search_type=line&search_term=Htr2a-Cre_KM207)  Expression in layers 5 and 6b | (+) | - | - | String projection |
| ORBm, FRP, MOs,PL, ILA, ORBvl | 297892843 | [Chrna2-Cre_OE25](http://connectivity.brain-map.org/transgenic/search?search_type=line&search_term=Chrna2-Cre_OE25)  Enriched in layer 5 | - | - | - | Small injection, string-like descending projection |
| **ORBVl** | 287769286 | [Rbp4-Cre_KL100](http://connectivity.brain-map.org/transgenic/search?search_type=line&search_term=Rbp4-Cre_KL100)  Enriched in layer 5 | + | +  dorsomedial | ++ | Nice specimen  Gemini |
| **ORBvl**, MOs, ACAd, PL, ILA, ORBl, ORBm | 167902586 | [Rbp4-Cre_KL100](http://connectivity.brain-map.org/transgenic/search?search_type=line&search_term=Rbp4-Cre_KL100)  Enriched in layer 5 | + | + dorsomedial | + | Very large injection, clearly transgressing the boundaries of VLO, Gemini |
| ORBvl, ILA, ORBl | 158435116 | C57BL/6J  WT | - | - | (+) | Slender, in thalamus only Sub. Extensive cortical projections |
| ORBVl, ORBm | 266564027 | Only layer 2-3 | - | - | - | Goes until striatum, no further |

Suppl. Table 1: ABA-data appertaining to 24 experiments in which tracers were injected into the LO-or the VLO- portion of the orbitofrontal cortex. With the exception of six wild–type (C57/BI6) mice, all others were genetically modified to express Cre-recombinase in specific sub-populations of neurons or cortical layers. The nomenclature corresponds to that utilized in the ABA. Translation to the (Franklin and Paxinos, 2008) abbreviations are as follows : ORBl = LO-cortex; ORBvl = VLO-cortex; ORVmN= MO; MOs = M2; Mop = M1; FRP = FrA cortex; ILA = IL-cortex; ACAd: Cg1; PL= PrL.
