# Supplementary figures and images for: The orbitofrontal cortex projects to the parvafox nucleus of the ventrolateral hypothalamus and to its targets in the ventromedial periaqueductal grey matter
Source: Brain Struct Funct. 2018 Oct 12;224(1):293–314. doi: 10.1007/s00429-018-1771-5 (PMC6373537; doi:10.1007/s00429-018-1771-5)

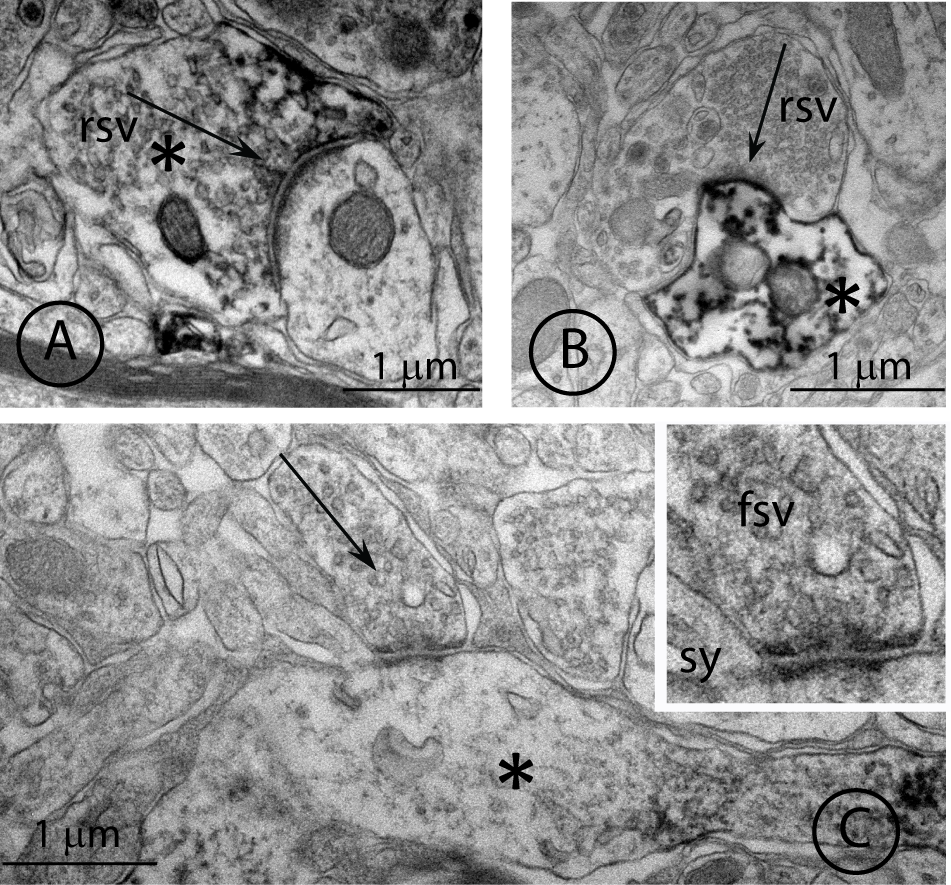

Supplement: Supplementary file 2 — Supplementary material 2 (TIF 1894 KB) [file 429_2018_1771_MOESM2_ESM.tif]

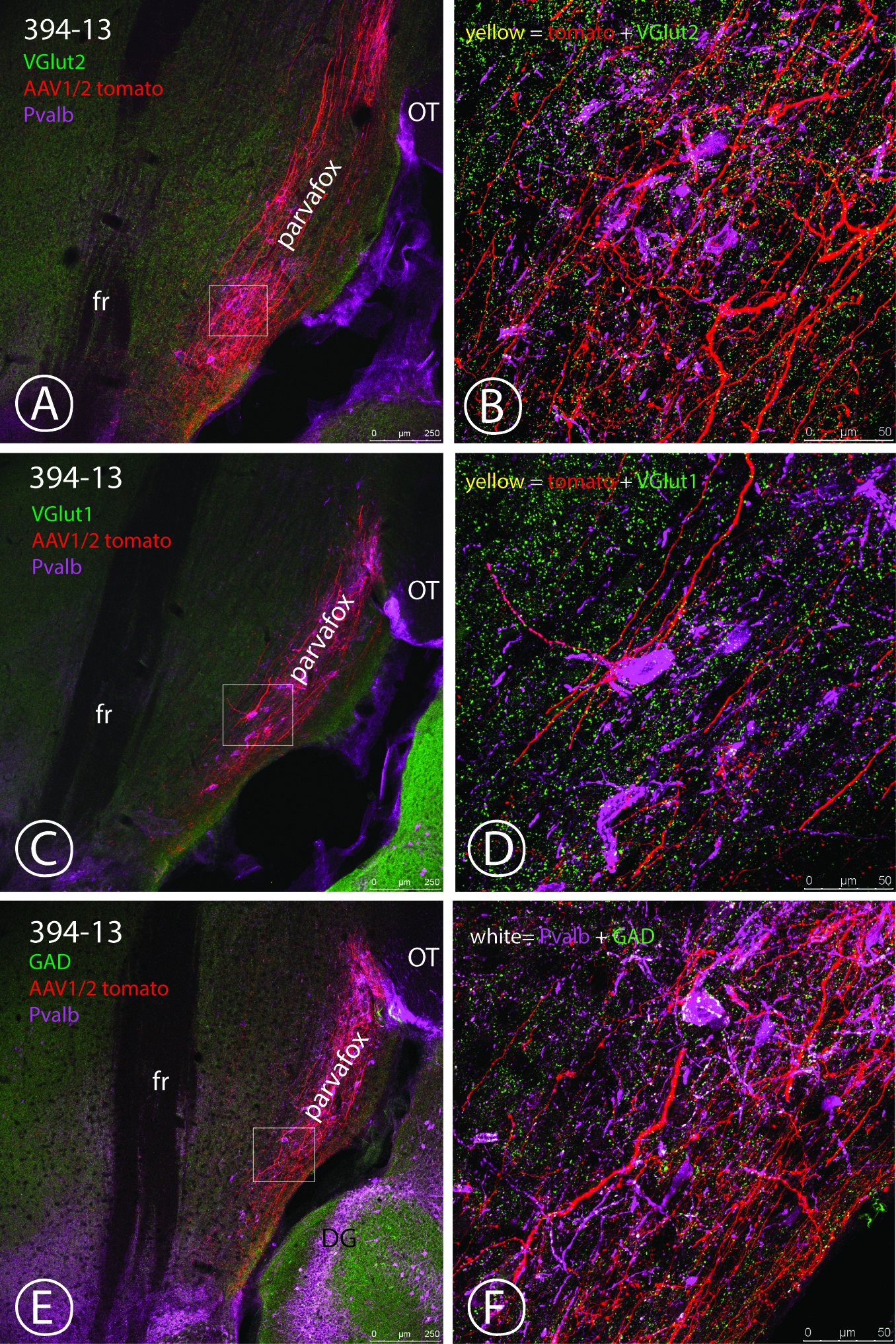

Supplement: Supplementary file 3 — Supplementary material 3 (TIF 12517 KB) [file 429_2018_1771_MOESM3_ESM.tif]
