# Supplementary material for: The orbitofrontal cortex projects to the parvafox nucleus of the ventrolateral hypothalamus and to its targets in the ventromedial periaqueductal grey matter
Source: Brain Struct Funct. 2018 Oct 12;224(1):293–314. doi: 10.1007/s00429-018-1771-5 (PMC6373537; doi:10.1007/s00429-018-1771-5)
Supplement: Supplementary file 4 — Supplementary material 4 (DOCX 55 KB) [file 429_2018_1771_MOESM4_ESM.docx]

**Supplementary material**

Supplementary Results

In the rodent OFC, the position of the medial boundary between the LO- and the VLO-cortices is not generally agreed upon by all authors. In some publications, the VLO-cortex in rats straddles laterally to the olfactory notch (Groenewegen, 1988; Yoshida et al., 1992), whereas in others, it borders the olfactory notch medially (Krettek and Price, 1977; Reep et al., 1996). In Paxinos’ atlases of the rat (Wistar) and mouse (C57Bl/J6) brains, (Paxinos and Franklin, 2013; Paxinos and Watson, 2009), the VLO-cortex is not mapped, whereas in Swanson’s atlas of the Sprague-Dawley rat brain (Swanson, 2004) it is; and likewise in the Allen Brain Institute mouse brain atlas (C57Black/6) (Dong, 2008). The arguments that come into play for setting the position of the boundary between the LO- and the VLO-cortices medial to the olfactory notch are namely that the latter region has privileged connections with the submedius thalamic nucleus and has reciprocal connections with the visual association areas C2M and Oc2L (Ray and Price, 1992; Reep et al., 1996). The lateral boundaries of the LO-cortex, on the other hand, are generally agreed upon; they coincide with the lateral border of the upper lip of the rhinal fissure. In our schematic drawings (e.g. Fig. 1 and 2), the subdivision of the orbitofrontal cortex in LO and VLO cortex has been adopted.

The Su3- and the PV2 nuclei

The Su3 nucleus (Carrive and Paxinos, 1994), which has a length of 40 µm in mice, is located ventral to the aqueduct, at the edge of the PAG between Bregma levels -3.80 and -4.20 (Franklin and Paxinos, 2008). In rats, it is located between Bregma levels -6 and -7 (Paxinos and Watson, 2009). The Su3 nucleus, which receive a strong parv-positive terminal field from the parvafox nucleus (Bilella et al., 2016c; Celio et al., 2013), constitutes one of the triple-layered structures that mantles the oculomotor nucleus, being sandwiched between an NADH-immunoreactive cap (Su3C) and a parvicellular band of cells (3PC), which projects to the abducens nucleus (Van Bockstaele and Aston-Jones, 1992). The dorsomedial half of the Su3 nucleus, which the findings of our study revealed to be targeted by the VLO-cortex, projects to the rostral and the caudal ventrolateral medulla, namely, to the cardiovascular and the respiratory sites in the medulla oblongata (Chen and Aston-Jones, 1996; Ennis et al., 1997; Van Bockstaele et al., 1991). Although the ventrolateral portion of the Su3 nucleus is shown in our report to receive terminals from the LO-cortex (e.g. Fig 3M, 4A-C), the targets of its projections have not been identified.

The PV2 nucleus has been described only recently (Celio et al., 2013) as an elongated column of parv-expressing neurons, visible in the cross section of Fig. 4I. In mice, it has a length of approximately 40 μm and is located ventral to the aqueduct, at the edge of the PAG extending between Bregma levels -4.60 and -5.05 (Franklin and Paxinos, 2008). In rats, it is located between Bregma levels -7.50 and -8.50 (Paxinos et al., 2009). Rostrally, it occupies the most ventral portion of the lateral dorsal raphe nucleus (DRL) – although it is not itself catecholaminergic (Fig. 4H) – and the most lateral region of the posterodorsal raphe nucleus (PDR), arching over the boundary of the isthmic reticular formation (isRT). The PV2 nucleus receives afferences from the two sub-populations of nerve cells of which the hypothalamic parvafox nucleus is composed, namely, the Pvalb- (Celio et al., 2013) and the *Foxb1-*expressing neurons (Bilella et al., 2016a). No information appertaining to its other connections and functions has yet been forthcoming.

**Supplementary results**

**Connections that confirms, specify or describes differences between known projections of the LO-VLO cortex.**

**Cerebral cortex**

The projections from the LO/VLO cortex targeted the contralateral cortical regions homotypical to the injection site. The LO-cortex innervated the granular and the agranular portions of the insular area and the Par2 (S2-cortex), as has been reported also by others (Reep et al., 1996). It projected descending axons to layer III of the piriform cortex (Illig, 2005), as well as to the temporal sensory and the secondary motor cortices (Bedwell et al., 2015; Bedwell et al., 2014). Projections from the VLO-cortex extended to the visual and the temporal cortices (Fig. 7B and C).

**Basal forebrain**

Some of the labelled axons coursed ventrally to the piriform cortex, whilst others left the striatum in the directions of the HDB, the SI and the olfactory tubercle (Fig. 7A, C). Endings were observed in the HDB in the region of the basal nucleus and in the magnocellular preoptic nucleus. These findings confirm those of other tracer experiments involving the *Phaseolus vulgaris* leucoagglutinin (Gaykema et al., 1991): injection RG137, figure 8; (Berendse et al., 1992; Zaborszky et al., 1997), which was injected into the LO- and the VLO-cortices (their cases 001, 026 and 085 in Fig. 5).

**The caudatoputamen and olfactory sites**

Axons leaving the OFC ascended to the external capsule and there formed tiny fascicles that entered the rostral part of the caudatoputamen, travelling in a dorsolateral-to-ventromedial direction throughout its extent (Fig. 7A). Axons deriving from neurons in the VLO-cortex were located more medially than were those arising from neurons in the LO-cortex, which confirms the findings of others (Berendse et al., 1992; McGeorge and Faull, 1989). When the injection hit the LO-cortex, an extensive terminal field arising from these axons was apparent in the ventrolateral quadrant; when it hit the VLO-cortex the terminal field was located in the laterodorsal quadrant, as has been previously described (Berendse et al., 1992; McGeorge and Faull, 1989; Zaborszky et al., 1997). The terminals infiltrated the matrix of the caudatoputamen but not the patches. A less dense mirror-projection, involving axons that were passing through the lower third of the *Genu corporis callosi* was observed in the contralateral caudatoputamen. Terminals were also detected in the shell of the accumbens (Acbsh); less so in the core region of the same nucleus (Acbc) and in the ventral *Globus pallidus*. The axons then converged in the ventromedial sector of the internal capsule and continued their course in the *crus cerebri*.

**Diencephalon**

**Thalamus**

From the axons that are located in the ventromedial sector of the internal capsule in the diencephalon, collaterals ascended to the inferior thalamic peduncle. They delaminated in the dorsomedial direction, forming terminal fields in the ventromedial tip of the reticular nucleus, the anterior thalamic nuclei (VA, VM, VL), the dorsolateral portion of the RE, the ventral half of the submedius nucleus (for the LO-cortex), and after piercing the internal medullary lamina, the MDC, the caudatus/pallidus-strip, the intralaminar nuclei [medial central (CM), paracentral (PC), lateral central (CL)], the posterior- and for the VLO-cortex, the parafascicular nuclei (Fig. 7C) (Jones and Leavitt, 1974). The nucleus submedius was a preferred target of the orbitofrontal projections from the VLO-, as has been already recognized by other authors (Coffield et al., 1992; Craig et al., 1982; Meyer, 1949; Yoshida et al., 1992). Only the ventral half received LO-derived terminals.

Although labelling in the mediodorsal nucleus was most intense in the central subnucleus, terminals were nevertheless always observed in the lateral and the medial segments. The thalamic field of terminals in the magnocellular portion of the MD corresponds to that of the LO- cortex in the thalamus (Uylings and van Eden, 1990). The relationship between the LO- cortex and the thalamus is reciprocal for the central segment of the mediodorsal nucleus, the ventral portion of the submedial nucleus and the ventromedial nucleus (Craig et al., 1982; Reep et al., 1996). Labelling of the thalamic nuclei was always bilateral and symmetrical, but was more intense on the ipsilateral side. A portion of the fibres gained access to the contralateral side via intrathalamic decussation.

**Amygdala**

At the same rostrocaudal level, a contingent of the labelled OFC- derived collaterals swerved ventrolaterally from the main corticofugal pathway to specifically innervate the lateral and the basolateral nuclei in the amygdala (Bregma level: 0.94; Fig. 7C), particularly when the injections encroached on the LO- or the insular cortex (AiD and AiV). The bodies of the nerve cells in the basolateral amygdala were also labelled (not shown), which is a rare but not an unknown occurrence (Chamberlin et al., 1998) of a retrograde-transport of the AAV-virus. This phenomenon can also be observed in the ABA-injections (e.g. image 64 of the injection Nr. 112306316).

**Glutamate and GABA in the OFC axons targeting the parvafox**

The identity of the neurotransmitter that is expressed by the LO/VLO-cortex-derived terminals in the parvafox nucleus and by those in the PAG was revealed immunohistochemically after exposing the specimens to antibodies against various vesicular glutamate transporters (Vglut1, Vglut2) and enzyme (GAD). Three-dimensional reconstructions of images that were recorded in the confocal scanning microscope revealed the primary neurotransmitter to be glutamate, which was retrieved in the synaptic vesicles by Vglut2 (suppl. Fig. 2 A, B). These findings correspond to those that have been recorded for terminals from the OFC to the basal forebrain (Zaborszky et al., 1997). But in addition, some of the terminals were revealed to use Vglut1 as a vesicular transporter (suppl. Fig. 2 C,D) or to express GAD (suppl. Fig. 2 E, F). The GAD-immunoreactive terminals may derive from the parvalbumin-expressing neurons in the LO-cortex, which contribute to the axonal projection to the parvafox nucleus. Recently, also glycine-expressing terminals probably deriving from the brainstem have been observed to abut on Glyt2–receptor–immunoreactive neurons in the parvafox nucleus (Szabolcsi et al., 2017).

**Supplementary Figures**

Supplementary Fig. 1:

LO/VLO terminals express mainly excitatory synapses

Ultrastructural study of contacts between terminals descending from the OFC and neurons in the region of the parvafox. The most common GFP-positive terminals were of the asymmetric type (marked with an arrow in panel A) and contained round synaptic vesicles (rsv). These synapses are known to be excitatory. The GFP-positive terminals (*) themselves, were targeted by both excitatory (arrow in B) or by symmetric, inhibitory synapses, characterized by presynaptic flat vesicles (fv; arrow in C; the inset shows a magnified view). These images were obtained on the brain of mouse 127-13. Scale bar: 1 μm.

Supplementary Fig. 2:

Neurotransmitters utilized by the LO/VLO projection

Co-existence of glutamatergic (VGlut1 or VGlut2) immunoreactivity or GABAergic (GAD immunoreactivity) markers in the terminal endings of the OFC-projection (labelled with AAV-tomato) in the parvafox nucleus.

Three horizontal sections through the level of the parvafox nucleus in the ventrolateral hypothalamus were immunostained with antibodies against the vesicular glutamate transporters Vglut 2 (panels A-C), VGkut 1 (panels C, D), respectively the GAD-enzyme (panel E, F). B, D and F show the framed area in A, C, D respectively under higher magnification. The yellow dots in the higher magnificataion images of panels B and D indicate the expression of Vglut1 or Vglut 2 in “boutons terminaux” or axons from the OFC. The white dots in F indicate the expression of GAD in Parv-positive terminals at the surface of Parv-neurons of the parvafox. These images were obtained with the brain of mouse 394-13. Scale bars: A, C, E: 250 μm; B, D, F: 50 μm.
